# Single‐Cell and Spatial Transcriptomic Profiling of Penile Squamous Cell Carcinoma Reveals Dynamics of Tumor Differentiation and Immune Microenvironment

**DOI:** 10.1002/advs.202500216

**Published:** 2025-06-05

**Authors:** Hongjian Song, Zhichao Tong, Guixiang Xie, Yaowei Li, Yubo Zhao, Fengpu Fan, Zongzheng Yang, Qing Shi, Qian Zhang, Feng Wen, Hongxue Meng, Haonan Li, Pengyu Guo, Dayong Hou, Zhenwei Zhang, Zhihao Yin, Ziyi Liu, Jianwei Wang, Peng Zhang, Peng Dai, Changfu Li, Lichen Teng, Yangyang Xu, Dexin Ding, Tao Xu, Jianzhang Li, Yongsheng Chen, Yu Qiu, XiaoWei Hu, Wentao Liu, Liang Wu, Roman Nawroth, Guibo Li, Hang Su, Ziqing Deng, Ziqi Wang, Di Wang, Wanhai Xu

**Affiliations:** ^1^ NHC Key Laboratory of Molecular Probes and Targeted Theranostics The Second Affiliated Hospital of Harbin Medical University, Harbin Medical University Cancer Hospital, Harbin Medical University Harbin 150001 China; ^2^ Department of Urology Harbin Medical University Cancer Hospital Harbin 150001 China; ^3^ Heilongjiang Provincial Key Laboratory of Basic Medical Sciences in Urology Cancer Harbin Medical University Cancer Hospital Harbin 150001 China; ^4^ Biobank Harbin Medical University Cancer Hospital Harbin 150001 China; ^5^ Department of Urogenital Medical Oncology Harbin Medical University Cancer Hospital Harbin 150001 China; ^6^ Department of Urology Klinikum rechts der Isar Technical University of Munich 80333 Munich Germany; ^7^ BGI Research Chongqing 401329 China; ^8^ Department of Urology Second Affiliated Hospital of Harbin Medical University Harbin 150001 China; ^9^ Center for Biomedical Materials and Engineering Harbin Engineering University Harbin 150001 China; ^10^ BGI Research Beijing 102601 China; ^11^ College of Life Sciences University of Chinese Academy of Sciences Beijing 100049 China; ^12^ Department of Pathology Harbin Medical University Cancer Hospital Harbin 150001 China; ^13^ Department of Medical Image Center Harbin Medical University Cancer Hospital Harbin 150001 China; ^14^ BGI‐Heilongjiang Omics Health Axis Intelligent Laboratory (BGI‐HL OHA Intelligent Laboratory) Harbin 150001 China; ^15^ Shanxi Medical University – BGI Collaborative Center for Future Medicine Shanxi Medical University Taiyuan 030001 China; ^16^ Department of Cystoscope Center Harbin Medical University Cancer Hospital Harbin 150001 China; ^17^ State Key Laboratory of Frigid Zone Cardiovascular Diseases Harbin Medical University Harbin 150001 China

**Keywords:** penile squamous cell carcinoma, single‐nucleus RNA sequencing, spatial landscape, spatial transcriptomics, tumor microenvironment

## Abstract

Penile squamous cell carcinoma (PSCC) is a highly aggressive malignancy without effective treatment due to limited knowledge of its development and tumor microenvironment (TME). In this study, single‐nucleus RNA sequencing (snRNA‐seq) and high‐resolution spatial transcriptomics are employed to comprehensively investigate the development trajectories and the TME. The results revealed that PSCC cells mimicked the differentiation and tissue organization of normal penile epithelium, independent of the human papillomavirus (HPV) infection status. Notably, a spatial subtype, Tum_1, appeared at early stage of tumor differentiation and in tumor–normal boundary regions. This subtype exhibited enhanced basal‐like and stemness features and showed high LAMC2 expression, which activated laminin‐integrin signaling via ITGA6/ITGB4, promoting tumor invasiveness. Furthermore, the results indicated that HPV‐positive basal stem‐like neoplasms dampened the immune function of T cells and macrophages, promoting an immunosuppressive environment that facilitates tumor progression. Supporting this, the patients with head and neck squamous cell carcinoma and lung squamous cell carcinoma who have high expression of HPV‐positive Tum_1 signatures derived greater benefit from PD‐1 blockade therapy. In summary, the findings provide a comprehensive spatial landscape of the PSCC TME and suggest potential treatment approaches targeting laminin‐integrin interaction and immunotherapy, especially in HPV‐positive patients.

## Graphical abstract

This study uses single‐nucleus RNA sequencing and spatial transcriptomics to investigate penile squamous cell carcinoma (PSCC). It reveals that PSCC tumor cells mimic normal penile epithelium differentiation, independent of HPV status. The Tum_1 subtype shows basal stem‐like characteristics and promotes invasiveness. HPV‐positive basal stem‐like tumors exhibit an immunosuppressive microenvironment, suggesting potential for personalized treatments based on HPV and tumor immune microenvironment.

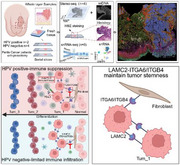

## 1 Introduction

Penile squamous cell carcinoma (PSCC) is a relatively rare, highly lethal malignancy that significantly impacts both survival and quality of life.^[^ ^1^, ^2^ ^]^ Radical surgical treatments for primary invasive disease, while achieving up to 81% 5‐year relative survival, often have profound physical and psychosexual consequences, including alterations in sexual and urinary function.^[^ ^3^, ^4^, ^5^ ^]^ In contrast, patients with advanced disease—such as those with positive pelvic or bilateral lymph nodes—have a significantly lower 5‐year relative survival of only 16%.^[^ ^6^ ^]^ Consequently, patients with positive lymph nodes are recommended to undergo neoadjuvant chemotherapy (NAC), with responders showing improved outcomes and a mean 5‐year survival of 56.9% following surgical consolidation.^[^ ^5^, ^7^ ^]^ Human papillomavirus (HPV) infection is a major etiological factor in PSCC, with the disease divided into HPV‐positive and HPV‐negative subtypes, each contributing approximately equally to the overall burden. Penile intraepithelial neoplasia (PeIN), the precursor lesion to penile cancer, is almost universally HPV‐driven, with up to 98.6% of PeIN 1–2 and 80.5% of PeIN 3 lesions testing HPV‐positive.^[^ ^1^, ^8^, ^9^ ^]^ Importantly, clinical studies have demonstrated that HPV‐positive PSCC is associated with better survival outcomes compared to HPV‐negative cases, likely due to differences in oncogenic pathways and tumor immune microenvironment (TIME) characteristics between the two subtypes.^[^ ^10^, ^11^ ^]^

The development of penile squamous cell carcinoma (PSCC) is driven by distinct molecular pathways depending on HPV status. The HPV‐positive PSCC is a multifactorial process, heavily influenced by persistent HPV infection, which triggers a cascade of molecular events culminating in malignant transformation. Chronic HPV exposure leads to the integration of viral DNA into the host genome, transforming basal epithelial cells into malignant phenotypes. HPV infection typically occurs through micro‐abrasions, with viral entry facilitated by specific receptors such as heparan sulfate proteoglycans and α6 integrin. Once integrated, HPV oncoproteins E6 and E7 play central roles in carcinogenesis.^[^ ^11^ ^]^ E7 inactivates the retinoblastoma protein (pRb), disrupting cell cycle regulation and enabling uncontrolled cell cycle progression. This disruption also interferes with the p16INK4A/pRb negative feedback loop, causing elevated p16INK4A expression, which serves as a reliable marker of HPV‐related PSCC. In fact, p16INK4A is highly expressed in 79.6% of HPV‐positive PSCC cases, compared to just 5% in HPV‐negative cases, making it a valuable surrogate biomarker for HPV infection. The E6 oncoprotein further contributes to carcinogenesis by promoting the degradation of p53, leading to the accumulation of genetic mutations and chromosomal instability. Additionally, E6 activating telomerase, and in combination with E7, promotes cellular immortality.^[^ ^12^, ^13^ ^]^ HPV‐negative tumors are often associated with genetic mutations such as TP53 mutations and alterations in tumor suppressor genes, which drive malignant transformation independent of viral integration. In contrast to the well‐defined pathogenesis of HPV‐driven cancers, HPV‐negative PSCC shows a more variable progression, often linked to environmental risk factors such as smoking or chronic irritation.

Our study provides a comprehensive molecular characterization of PSCC using snRNA‐seq and high‐resolution spatial transcriptomics to map the tumor's differentiation progression and its interaction with the TIME.^[^ ^14^, ^15^ ^]^ We found that PSCC tumor cells mimic the differentiation trajectory and tissue organization of normal penile epithelium, independent of HPV infection status. Specifically, we identified the Tum_1 tumor cell subtype, which is present in the early stages of tumor differentiation and in the intermediary regions adjacent to normal tissues. Tum_1 cells exhibited heightened basal‐like and stemness characteristics, with increased activation of key oncogenic pathways, including stem cell proliferation and differentiation, integrin binding, and laminin interactions. Notably, the Tum_1 subtype showed elevated expression of LAMC2, which facilitated tumor invasiveness through its interaction with ITGA6/ITGB4.^[^ ^16^, ^17^ ^]^

Furthermore, our results revealed significant differences in the immune microenvironment between HPV‐positive and HPV‐negative PSCC. Our analysis revealed that T cells in close proximity to malignant cells from HPV‐positive tumors exhibited significantly higher inhibitory scores. This suggests that HPV status may dampen the immune function of T cells, possibly promoting an immunosuppressive environment that could facilitate tumor progression. Similarly, macrophages adjacent to malignant cells from HPV‐positive tumors displayed reduced pro‐inflammatory scores, which indicated a shift towards a more immunosuppressive phenotype. These findings underscore the molecular and immune landscape differences between HPV‐positive and HPV‐negative PSCC, highlighting the potential for more personalized treatment strategies based on HPV status and the TME.

## 2 Results

### 2.1 High‐Resolution Single‐Cell and Spatial Transcriptomic Profiling Uncovers the Cellular Architecture and TME in PSCC

**Figure 1:**
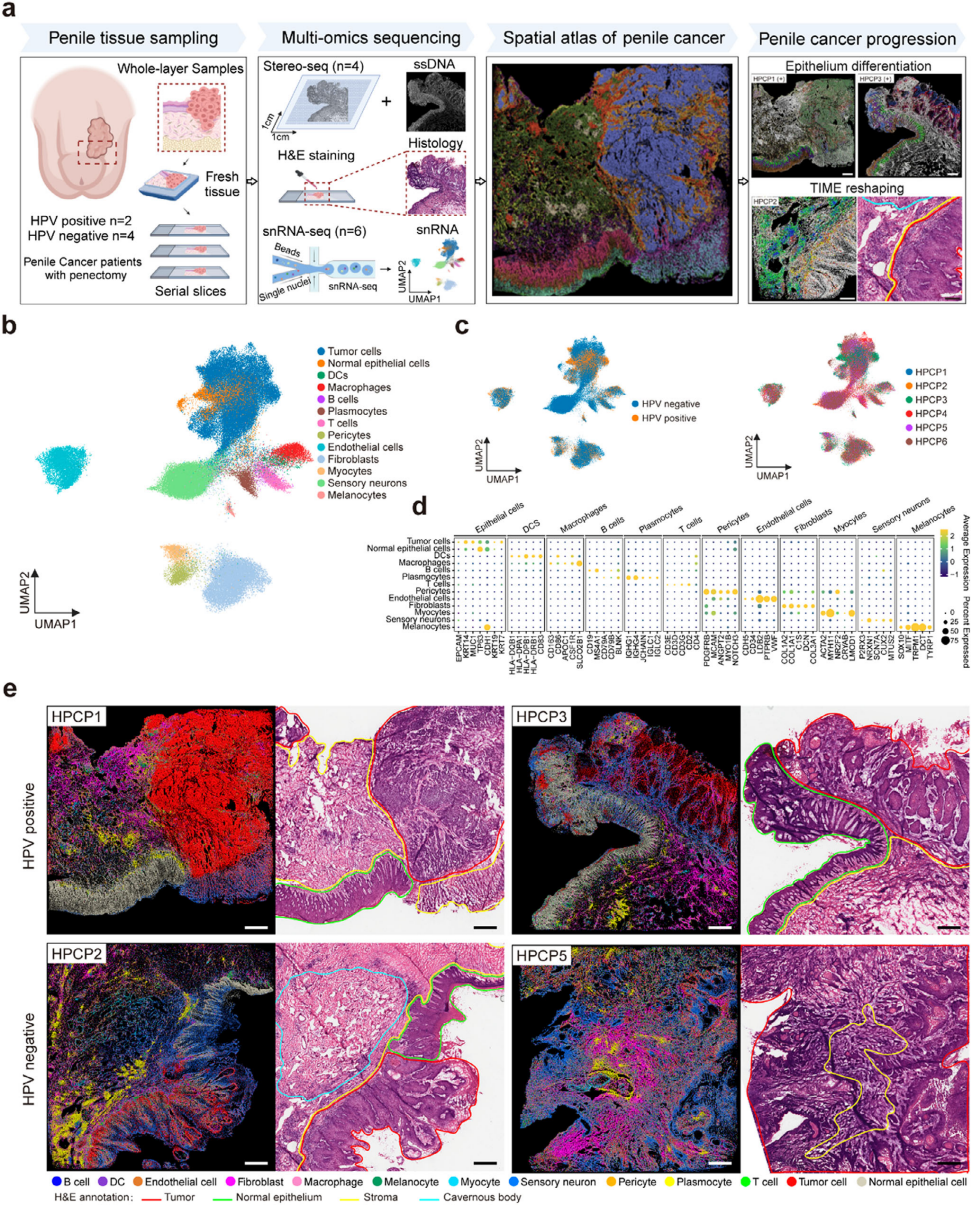
Comprehensive single‐cell and spatial transcriptomic atlas of penile cancer. a) Schematic overview of the integrated spatial omics design and workflow. The spatial transcriptomics (Stereo‐seq, four samples) and single‐nucleus RNA sequencing (snRNA‐seq, six samples) data were acquired from six patients (two patients with HPV infection and four patients without HPV infection). Integrative data analysis revealed the mechanism of penile cancer occurrence and progression, and immune microenvironment reshaping related to HPV infection status. (Created in BioRender. BioRender.com/e56j400). b) UMAP visualization of the snRNA‐seq dataset, where cells are color‐coded based on their respective cell subtypes. c) UMAP visualization showing the organization of snRNA‐seq data by HPV infection status (left) and individual patient identity (right). On the left, cells are grouped and color‐coded to distinguish HPV‐positive and HPV‐negative cases, revealing transcriptomic differences driven by HPV infection status. On the right, cells are grouped by individual patients. d) Dot plot illustrating the expression patterns of key marker genes across identified cell types within the snRNA‐seq data. Each dot represents a gene's expression in a specific cell type, with the color intensity indicating the average expression level and the dot size reflecting the percentage of cells within that cell type expressing the gene. e) Spatial mapping of annotated single‐cell types onto spatial transcriptomic data derived from tissue sections of two HPV‐positive and two HPV‐negative patients. The spatial distribution of cell types is overlaid onto tissue sections, allowing the visualization of cell populations within their native spatial context. Adjacent pathological hematoxylin and eosin (H&E)‐stained images provide histological reference, with key tissue regions annotated and color‐coded: tumor regions outlined in red, normal epithelium regions in green, and stromal regions in yellow. This spatial mapping emphasizes the characteristic states of cell type distribution and the tumor microenvironment in penile cancer tissues.

To investigate the spatial organization and the underlying mechanisms driving microenvironment remodeling in PSCC, we obtained tumor tissue samples from six patients, which included two HPV‐positive and four HPV‐negative cases. We conducted snRNA‐seq on all samples, complemented by spatial transcriptomics at single‐cell resolution (Stereo‐seq) on four samples (**Figure** **1**a; and Figure S1a, (Supporting Information) and Table S1, (Supporting Information).^[^ ^18^ ^]^ The snRNA‐seq data yielded 96904 high‐quality cells, which were subsequently clustered and annotated. Thirteen primary cell types were identified based on signature genes, including dendritic cells (*HLA‐DPB1*, *CD83*), macrophages (*CSF1R*, *SLCO2B1*), B cells (*MS4A1*, *CD19*), plasma cells (*IGHG1*, *IGHG4*), T cells (*CD2*, *CD3G*), pericytes (*PDGFRB*, *MCAM*), endothelial cells (*VWF*, *PTPRB*), fibroblasts (*COL1A1*, *COL1A2*), myocytes (*ACTA2*, *MYH11*), sensory neurons (*NRXN1*, *CUX2*, and *MTUS2*), and melanocytes (*MITF*, *TRPM1*, and *TYRP1*).^[^ ^19^ ^]^ In addition, epithelial cells were classified into normal and tumor populations using inferCNV analysis (Figure 1b–d; and Figure S1b–d, Supporting Information).^[^ ^20^, ^21^ ^]^ These cell types were consistent with previous studies on penile cancer, with the exception of sensory neurons,^[^ ^22^ ^]^ which were likely detected due to the snRNA‐seq's ability to capture nerve‐associated cells.^[^ ^23^, ^24^ ^]^ The spatial transcriptomics data consisting of 1785392 cells were further integrated, allowing spatial mapping of the snRNA‐seq data (see methods, Figure 1e).^[^ ^25^ ^]^ The spatial distribution of cell types closely mirrored the pathological regions identified by experienced pathologists in H&E‐stained images (Figure 1e). For example, tumor cells are predominantly localized to the tumor tissue area, normal epithelial cells to normal tissue regions, and fibroblasts to the stromal areas (Figure 1e).

### 2.2 PSCC Tumor Cells Mimic a Stratified Differentiation Trajectory with Distinct Spatial and Molecular Features Compared to Normal Epithelium

**Figure 2:**
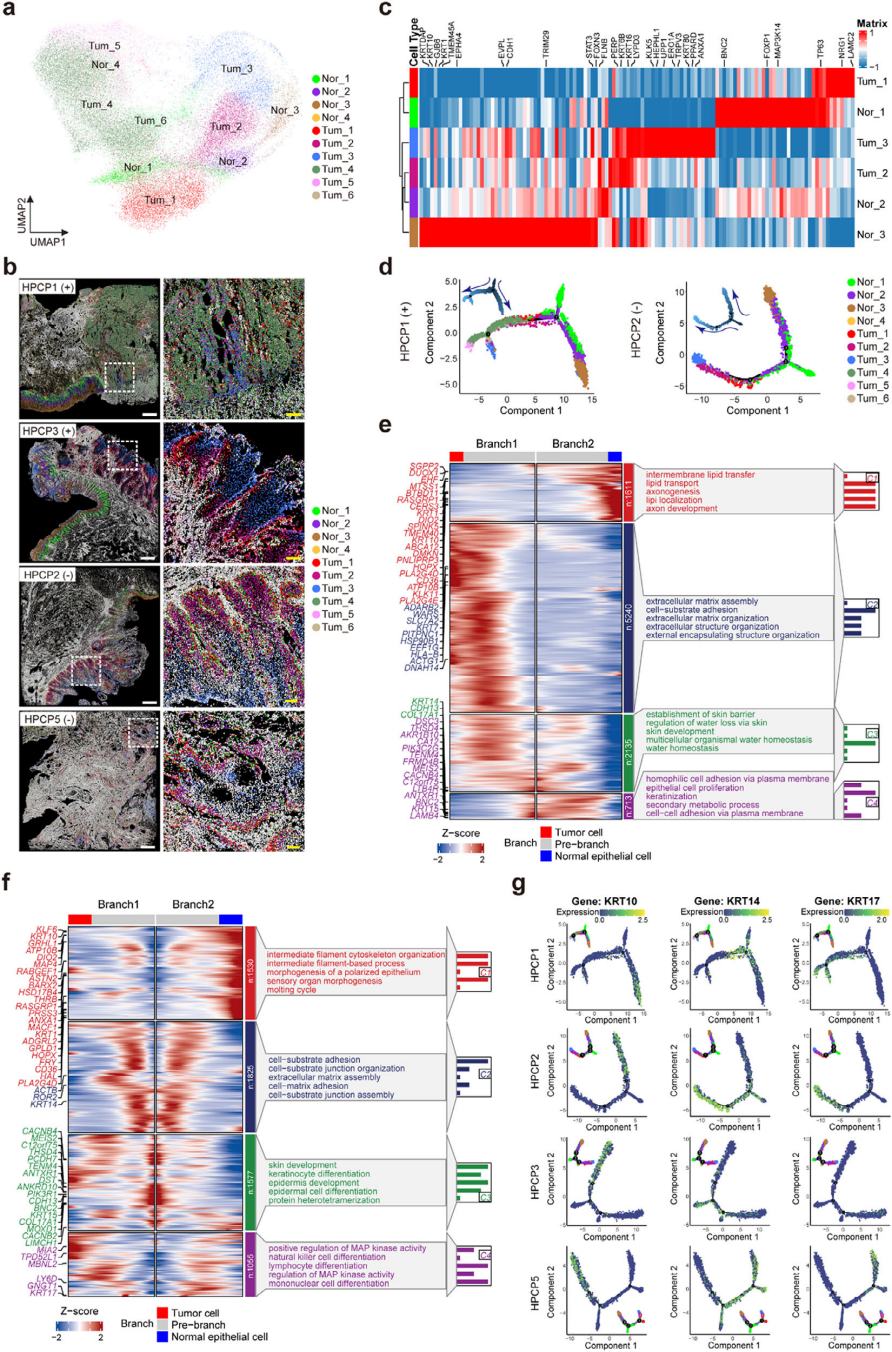
Differentiation inference of normal epithelium and tumor cells. a) UMAP visualization of the tumor cells and normal epithelial cells in the snRNA‐seq dataset by employing unsupervised clustering, where cells are color‐coded based on their respective subtypes. b) Spatial distributions of normal epithelial cell subtypes and tumor cell subtypes for samples HPCP1, HPCP2, HPCP3, and HPCP5, determined by the Spoint deconvolution method. Samples that test positive for HPV are denoted with a “(+)”, while those that test negative for HPV are marked with a “(‐)”. The boxed areas were identified as tumor cell subtypes for further magnification on the right, depicting the spatial characteristics of tumor cell subtypes. White scale bar, 1 mm; Yellow scale bar, 200 µm. c) Heatmap with unsupervised clustering illustrating the expression patterns of marker genes across three distinct normal epithelial cell subtypes (subtypes 1, 2, and 3) as well as three tumor cell subtypes (subtypes 1, 2, and 3). d) The pseudo‐temporal trajectory among epithelial cell subtypes within the context of HPV infection status is illustrated, distinguishing between HPCP1 (HPV‐positive) and HPCP2 (HPV‐negative) samples. This visual representation employs a pseudo‐temporal path to trace the developmental progression of these cell subtypes. Arrows strategically positioned around the pseudo‐temporal path serve as guides, delineating the directionality of cellular evolution over time. e, f) Pseudo‐temporal branch analysis between the two trajectory paths to normal epithelial cells and tumor cells, respectively, for HPCP1 sample (e) and HPCP2 sample (f). On the left side of the visualization, gene names are annotated to identify the specific markers associated with each branch of the trajectory. The right side is dedicated to annotating enrichment pathways, which represent the biological processes along the pseudo‐temporal trajectory. g) The expression dynamics of key marker genes *KRT10*, *KRT14*, and *KRT17* along the trajectory path of all samples. Zoomed‐out trajectory plot is labeled by epithelial cell subtypes.

To explore the specific mechanisms driving the development and evolution of PSCC, we performed subtype analysis on 5948 normal epithelial cells and 36492 tumor epithelial cells, identifying four normal epithelial subtypes and six tumor epithelial subtypes (**Figure** **2**a; Figure S2a–c, Supporting Information). Spatial mapping subsequently showed that three normal epithelial subtypes—Nor_1, Nor_2, and Nor_3—correspond to the basal layer, stratum spinosum, and stratum corneum regions of the normal penile epithelium (Figure 2b; Figure S2d, Supporting Information).^[^ ^26^, ^27^, ^28^, ^29^ ^]^ Gene expression analysis further confirmed that these cell types exhibited characteristic gene expression profiles, with Nor_1 cells enriched for basal cell markers (*TP63*, *FOXP1*), Nor_2 for spinous cell markers (*FLNB*, *KRT1*, and *KRT10*), and Nor_3 for superficial cell markers (*EVPL*, *CDH1*) (Figure 2c). Intriguingly, several tumor epithelial subtypes also displayed specific spatial distributions, with Tum_1, Tum_2, and Tum_3 showing stratified patterns reminiscent of Nor_1, Nor_2, and Nor_3, respectively (Figure 2b). Notably, Tum_1 demonstrated a gene expression profile closely resembling Nor_1, expressing high levels of *TP63* and other basal markers, yet also uniquely overexpressing genes such as *LAMC2* and *NRG1*. Tum_2′s profile was similar to that of Nor_2, co‐expressing *FLNB*, but uniquely overexpressing *KRT6B* and *KRT16*. Tum_3 resembled Nor_3, co‐expressing *CDH1* and *STAT3*, but also overexpressing *KLK5*, *KRT80*, and *ANXA1* (Figure 2c). These findings suggest that PSCC tumor cells undergo a differentiation process parallel to the normal penile epithelium but with distinct gene expression and regulatory changes.

To further investigate the differentiation trajectories of tumor epithelial cells, we conducted pseudotime analysis on the epithelial cells from each sample.^[^ ^30^ ^]^ Interestingly, the results revealed two distinct differentiation paths, one corresponding to normal epithelial differentiation and the other to tumor epithelial differentiation, irrespective of HPV infection status (Figure 2d; Figure S2e, Supporting Information). Tracing the temporal progression, both branches began from Nor_1, with one path progressing through Nor_2 and Nor_3, while the other branched into Tum_1, Tum_2, and Tum_3. Gene and function analyses along these two pathways revealed distinct functional expressions: normal epithelial differentiation was associated with terminal differentiation into keratinocytes, intermediate filament‐based processes, and lipid transport, while tumor differentiation was linked to increased cell‐substrate adhesion, extracellular matrix assembly, and activation of the MAPK and RAS signaling pathways (Figure 2e,f; Figure S2f,g, Supporting Information). Furthermore, temporal variation in keratin expression differed between normal and tumor cells, with *KRT10* highly expressed exclusively during normal epithelial differentiation, while *KRT17* was highly expressed throughout tumor progression (Figure 2g). We also found that transcription factor *RUNX1* and gene *PITPNC1* were highly expressed along the trajectory to tumor cells but not on the path to more differentiated epithelial cells. This suggests that *RUNX1* and *PITPNC1* may play significant roles in the progression of PSCC tumors (Figure S2h, Supporting Information). In conclusion, while the developmental process and tissue organization of PSCC tumor cells resemble those of normal penile epithelium, the regulatory networks driving these processes are distinctly altered.

### 2.3 Basal‐Like Tumor Cell Subpopulation Tum_1 Exhibits High Invasiveness and Stem‐Like Differentiation Features

**Figure 3:**
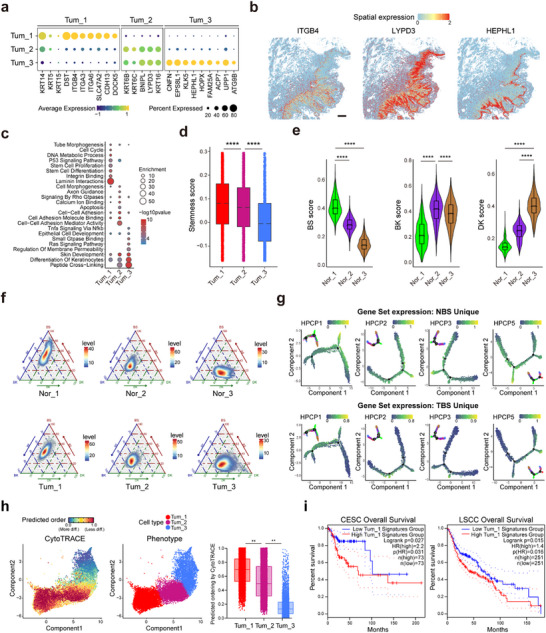
Tumor cells retain epithelial‐like tissue differentiation structures. a) Dot plot showing gene expression profiles specific to tumor subtypes 1, 2, and 3. The color intensity indicates the average expression level of genes, while the dot size reflects the percentage of cells within that subtype expressing the gene. b) Spatial gene expression of *ITGB4*, *LYPD3*, and *HEPHL1* in the Stereo‐seq data, highlighting potential tissue‐level organization features. c) Dot plot illustrating functional enrichment pathways of subtype‐specific genes for tumor subtypes 1, 2, and 3. The plot uses the ‐log10(p‐value) to signify the statistical significance of each pathway and enrichment score to quantify the extent of pathway enrichment. d) Stemness scores for tumor subtypes 1, 2, and 3 in snRNA‐seq data, statistically tested using the Wilcoxon test with Benjamini‐Hochberg correction. e) Violin plot validating epithelial feature scores against the distribution of normal epithelial cell subtypes identified in snRNA‐seq analysis. The analysis uses the Wilcoxon test with Benjamini‐Hochberg correction. BS: basal stem; BK: basal keratinocyte; DK: differentiated keratinocyte. f) Ternary plot comparing the phenotypic similarities and differences between tumor epithelium and normal epithelium. This plot provides a visual comparison of the phenotypic characteristics, highlighting both the shared and distinct features between the tumor and normal epithelium. g) Monocle2 analysis of the evolutionary trajectories of tumor‐ and normal‐epithelium‐specific BS gene expression patterns. The expressions of normal BS (above) and tumor BS (below) gene sets on the trajectory path of epithelial subtypes are shown for all samples. Zoomed‐out trajectory plot is labeled by epithelial cell subtypes. h) CytoTrace analysis depicting the differentiation order of tumor cell subtype clusters. Cumulative box plots showing the predicted differentiation ordering scores across tumor cell subtypes, statistically tested using the Wilcoxon test with Benjamini‐Hochberg correction. i) Survival analysis of high and low expression of Tum_1 signature genes in CESC and LSCC patients from the TCGA cohort. These survival curves show the prognostic value of Tum_1 subtype signature genes, statistically tested using the Mantel‐Cox log‐rank test. Statistical significance is indicated by ****p < 0.0001.

To identify functional differences among tumor cell subtypes, we conducted comparative analyses. Differential gene expression analysis demonstrated that Tum_1 tumor cells predominantly expressed basal cell marker genes such as *KRT14*, *KRT5*, and *KRT15*, and exhibited high levels of genes related to cell adhesion, including *ITGB4* and *ITGA6*. In contrast, Tum_3 tumor cells primarily expressed genes linked to epithelial differentiation and keratinization, such as *CNFN*, *EPS8L1*, and *HEPHL1* (**Figure** **3**a,b). Functionally, Tum_1 cells exhibited increased P53 signaling, stem cell proliferation and differentiation, integrin binding, and laminin interactions (Figure 3c). Stemness scoring revealed that Tum_1 displayed the highest stem‐like properties, while Tum_3 exhibited the lowest (Figure 3d). Previous studies have identified three functional subtypes within the esophageal squamous epithelium: basal stem cells (BS), transiently proliferating basal keratinocytes (BK), and postmitotic differentiated keratinocytes (DK),^[^ ^31^ ^]^ each characterized by unique morphology, tissue spatial distribution, and molecular features. BS cells serve as the progenitors of BK and DK cells. Our spatial analysis indicated that Tum_1 cells were localized to the inner regions of the tumor, near the stromal components. Thus, we hypothesized that the penile squamous epithelium might exhibit similar structural and functional traits. To verify this, we assessed the expression of BS, BK, and DK markers across different epithelial cell subtypes using characteristic scoring. The results indicated a gradual decrease in BS marker expression from Nor_1 to Nor_2 to Nor_3, while DK marker expression increased. The Nor_2 epithelial subtype displayed the most pronounced BK marker expression, suggesting that penile squamous epithelial cells follow a similar structural composition and differentiation trajectory to esophageal squamous epithelium (Figure 3e; Figure S3a, Supporting Information). Although esophageal cancer cells have been shown to express all three epithelial cell gene signatures, our findings in PSCC revealed three distinct tumor cell subtypes with BS, BK, and DK marker distribution patterns that mirrored those seen in both tumor regions and normal epithelial areas (Figure S3a, Supporting Information). This led us to posit that PSCC tumor cells may also exhibit characteristics of various developmental stages. We thus isolated characteristic genes for the three normal epithelial subtypes and three tumor epithelial subtypes to investigate the traits of normal and tumor squamous epithelium at different differentiation stages (see Experimental Section, Figure S3b (Supporting Information) and Table S2, Supporting Information). The analysis revealed that both normal and tumor epithelia shared similar characteristic distributions according to their scoring, yet Nor_1 cells exhibited stronger BS characteristics than Tum_1, and Tum_3 tumor cells displayed more pronounced DK characteristics than Nor_3 (Figure 3f). This suggests that normal basal cells possess more robust basal stemness features, while tumor epithelial keratinization is more evident. Additionally, we analyzed the characteristic distributions of normal squamous epithelium and PSCC tumor squamous epithelium identified by our study and observed that their distribution patterns over time were consistent with epithelium developmental stages (Figure 3g; and Figure S3c, Supporting Information).

We further investigated the differentiation status and developmental potential of the three tumor subtypes using the CytoTRACE algorithm. Our analysis revealed that Tum_1 cells had the lowest degree of differentiation and possessed the highest developmental potential when compared to Tum_2 and Tum_3 (Figure 3h). This suggests that Tum_1 cells not only share basal stem cell‐like gene expression characteristics but also retain additional features typical of cancer stem‐like cells, such as the ability to self‐renew and differentiate into various tumor cell types. The high developmental potential of Tum_1 further confirms its role as a key player in tumor initiation and progression. To explore the relationship between the tumor subtypes and patient prognosis, we calculated the marker gene sets for each of the three tumor subtypes. We examined their expression correlations with overall survival in cervical squamous cell carcinoma and endocervical adenocarcinoma (CESC) and lung squamous cell carcinoma (LSCC) patients from the TCGA cohort. The results indicate that only the high expression of the marker gene set for Tum_1 subtype is significantly associated with poor prognosis (Figure 3i; Figure S3d, Supporting Information).

### 2.4 LAMC2 is Highly Expressed in Tum_1 and Promotes Tumor Progression

**Figure 4:**
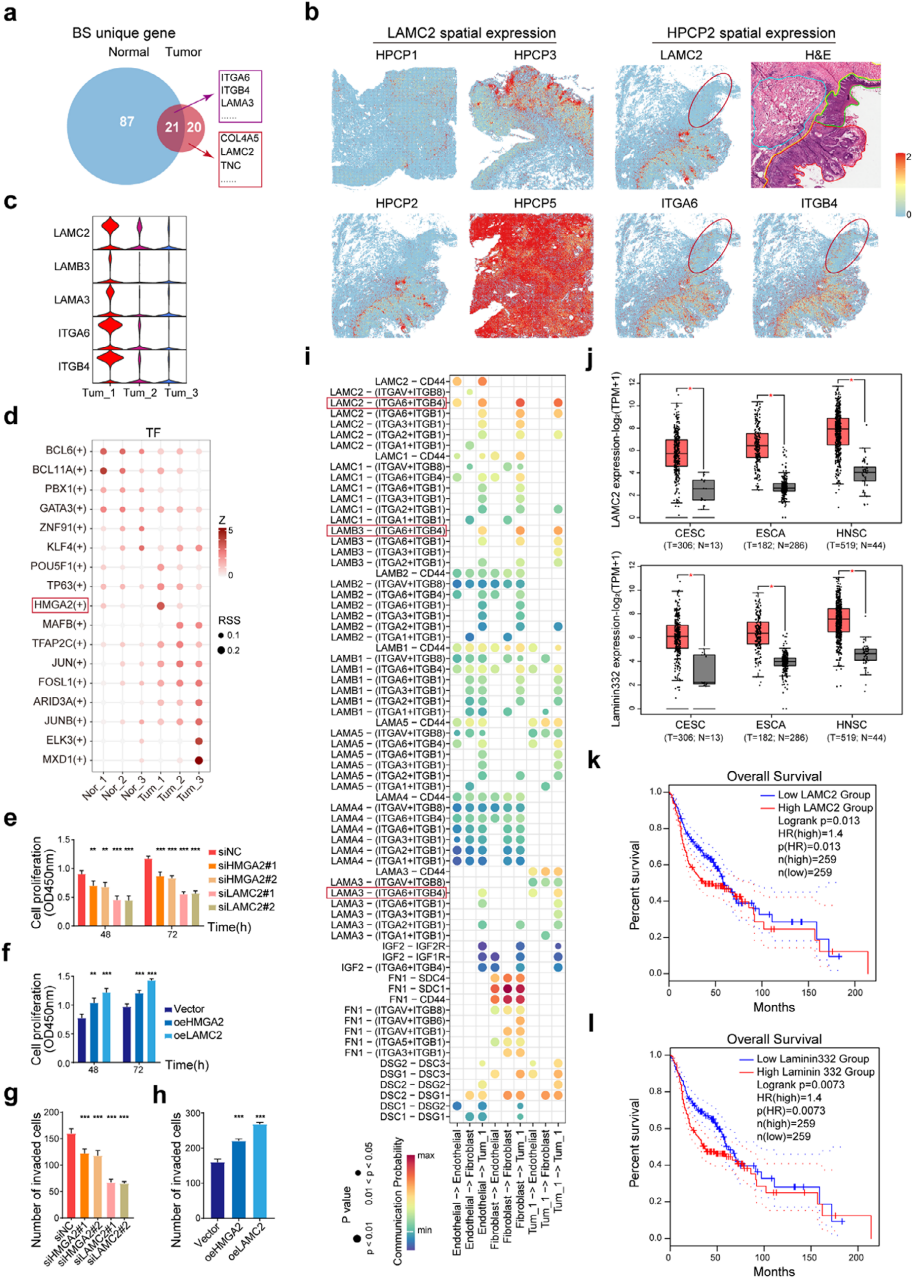
Two sources of Laminin‐332 promote the invasion of tumor basal stem‐like cells. a) Venn diagram illustrating the overlapping and distinct basal stem‐like cell signatures between tumor and normal epithelial tissues. The diagram highlights genes that are specific to tumor basal stem‐like cells, as well as the genes shared between tumor and normal basal stem‐like cells. Key representative genes for the intersection and tumor‐specific signatures are labeled. b) Quantitative expression patterns of Laminin‐332 subunit LAMC2 and its receptors (ITGA6/ITGB4) in spatial transcriptomics. Red ellipses on the spatial transcriptomics images emphasize the corresponding regions in the pathological tissue sections of normal epithelium. c) Violin plot validating the expression levels of Laminin‐332 subunits and the integrin families among the tumor cell subtypes. d) SCENIC transcription factor analysis across different cell layers in tumor and normal epithelial tissues. This analysis identifies key transcription factors that regulate gene expression in cell subtypes within the tumor and normal epithelium. e, f) Cell proliferation in Penl1 cells was evaluated after HMGA2 and LAMC2 knockdown (siHMGA2 and siLAMC2) or overexpression (oeHMGA2 and oeLAMC2) via small interfering RNA and plasmid transfection, respectively, for 48 and 72 h, statistically tested using the two‐tailed unpaired Student's t‐test. g, h) Transwell invasion assay was performed in Penl1 cells following HMGA2 and LAMC2 knockdown (siHMGA2 and siLAMC2) or overexpression (oeHMGA2 and oeLAMC2) via small interfering RNA and plasmid transfection for 48 h, statistically tested using the two‐tailed unpaired Student's t‐test. i) Ligand‐receptor interactions between tumor basal stem‐like cells and stromal cells. This analysis explores the molecular interactions involved in the communication between Tum_1 cells and the surrounding stromal cells. j) Bulk expression differences of *LAMC2* and Laminin‐332 subunit encoding genes in cervical, esophageal, and head/neck squamous cancers compared to normal tissues. Comparisons include data from TCGA normal samples and GTEx, illustrating the upregulation of these genes in various cancer types, statistically tested using the two‐sample t‐test. k, l) Survival analysis of high and low expression of *LAMC2* (k) and Laminin‐332 (l) subunit encoding genes in head/neck squamous cancer. These survival curves show the prognostic value of *LAMC2* and Laminin‐332 subunit encoding genes, statistically tested using the Mantel‐Cox log‐rank test. Statistical significance is indicated by ***p < 0.001, **p < 0.01, *p < 0.05.

To investigate the mechanisms underlying tumor progression, we compared gene expression profiles of normal basal stem‐like cells (Nor_1) and tumor basal stem‐like cells (Tum_1), which shared basal localization and stem cell characteristics (Figure 3e,f). Both cell types co‐expressed the integrin heterodimer ITGA6/ITGB4 (**Figure** **4**a), which has been linked to tumor metastasis via Laminin‐332‐mediated activation of mTOR and MAPK signaling pathways (Figure S3e, Supporting Information).^[^ ^32^, ^33^ ^]^ Notably, we found that *LAMC2*, encoding the γ2 chain of Laminin‐332, which is a ligand of the integrin heterodimer (ITGA6/ITGB4),^[^ ^34^ ^]^ was exclusively expressed and spatially localized to Tum_1 cells, with notably high expression in the T3 stage sample HPCP5 (Figure 4b,c). In addition, *LAMA3* and *LAMB3*, encoding the α3 and β3 chains of Laminin‐332, were also highly expressed in the tumor basal stem‐like cell region compared to normal epithelial areas (Figure S3f, Supporting Information). Next, transcription factor analysis using the SCENIC pipeline revealed that HMGA2, a key transcription factor known to regulate *LAMC2* expression in tumors, was significantly activated in Tum_1 cells, suggesting a role in promoting tumor basal stem‐like cell activity (Figure 4d). To validate our findings, we utilized RNA interference to silence HMGA2 and LAMC2 in the PSCC cell line Penl1. This intervention led to a significant reduction in LAMC2 expression, which was associated with a marked inhibition of cell proliferation, migration, and invasion. In contrast, overexpression of HMGA2 and LAMC2 resulted in elevated LAMC2 levels and enhanced cell proliferation, migration, and invasion. Notably, these effects were more pronounced when LAMC2 processing was modulated (Figure 4e–h; Figure S4a–d, Supporting Information). We also explored the interaction between stromal cells and tumor basal stem‐like cells and observed a strong communication probability between Laminin‐332 (via LAMC2) and ITGA6/ITGB4, both endogenously (between Tum_1 cells) and exogenously (from fibroblasts to Tum_1 cells), while FN1 signaling predominantly governed fibroblast interactions with other cell types, independent of integrin receptors (Figure 4i). To validate these findings, we analyzed data from TCGA on other squamous cell carcinomas (CESC, ESCC, HNSC) and observed that expression levels of *LAMC2* and Laminin‐332 genes were consistently higher in tumors than in normal tissues (Figure 4j). Survival analysis indicated that high expression of these genes were significantly correlated with reduced overall survival (OS) (Figure 4k,l). To further validate this conclusion, we performed immunohistochemistry analysis of LAMC2. The results demonstrated that the expression was significantly elevated in patients with recurrence within the validation cohort (Figure S4e, Supporting Information). These results suggest that Tum_1‐positive tumors may represent a higher‐risk subgroup in PSCC, which highlights the importance of focusing on Tum_1 (tumor basal stem‐like cells) when devising treatment strategies in future clinical practice. Taken together, these findings suggest that tumor basal stem‐like cells in PSCC may activate the ITGA6/ITGB4 signaling axis through both autocrine and paracrine pathways involving Laminin‐332, thereby promoting tumor invasion.

### 2.5 Different Tumor Cell Subtypes Reshape Tumor Immune Microenvironment

**Figure 5:**
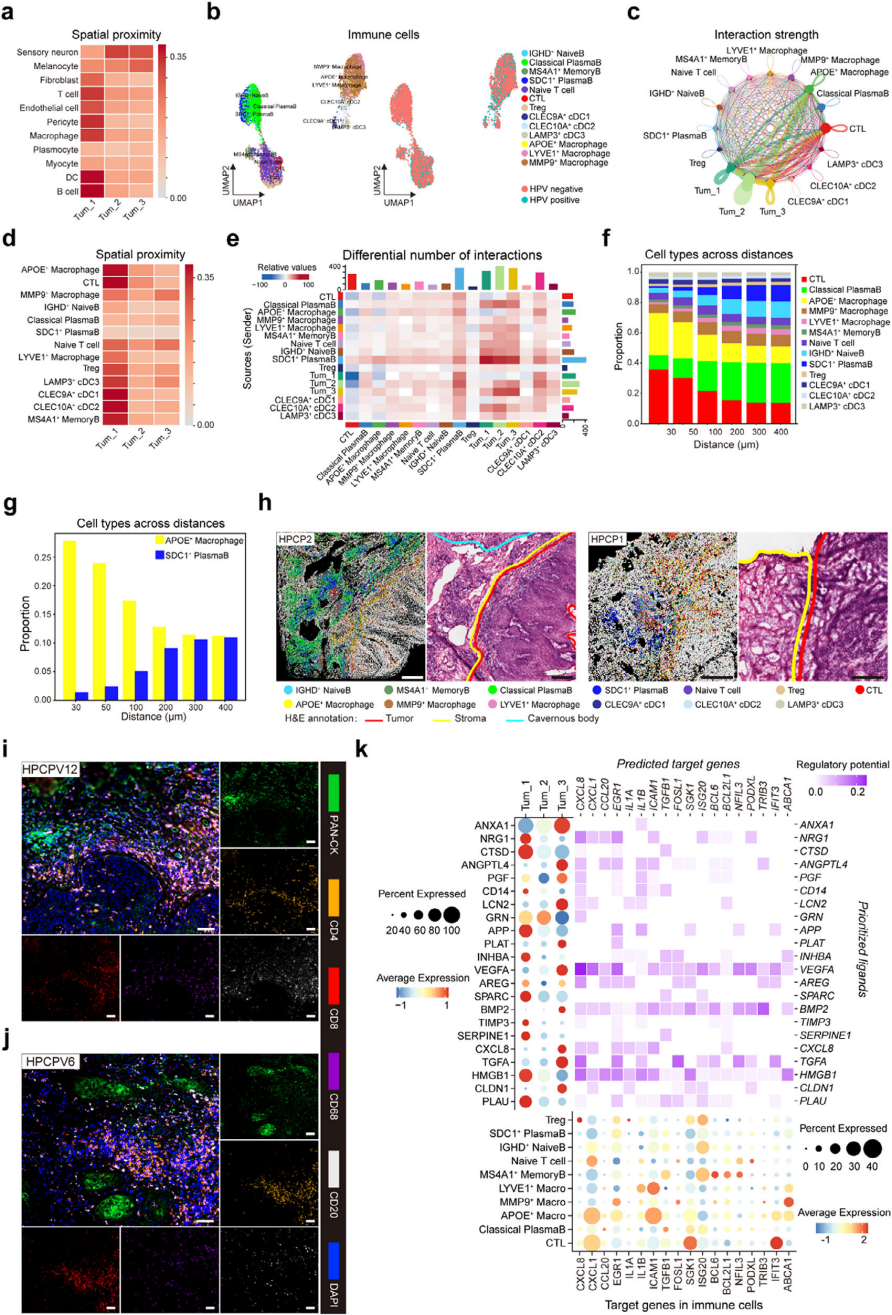
Immune landscape around tumor cells and cell communication regulation. a) Heatmap showing cell type proportions of being neighbors within the 30 um radius surrounding tumor cell subtypes 1, 2, and 3. b) Umap visualizations of immune cell subtypes, labeled by cell subtype and HPV infection status, respectively. c) Cell‐cell interaction strength between different cell types within the region of the 100‐um radius around Tum_1. The width of the line is represented as the interaction strength. d) Heatmap showing immune cell subtype proportions of being neighbors within the 30 um radius surrounding tumor cell subtypes 1, 2, and 3. e) Heatmap showing the differences in numbers of cell‐cell interactions when comparing the region of the 200 um radius around Tum_1 to the region of the 100 um radius around Tum_1. f) Stacked bar plot showing the immune cell subtype proportions of being neighbors within different radius distances surrounding Tum_1. g) The proportions of APOE^+^ macrophage and SDC1^+^ plasma cell of being neighbors within different radius distances surrounding Tum_1. h) Spatial distributions of immune cell subtypes, determined by the Spoint deconvolution method. Adjacent pathological HE‐stained images highlight key tissue regions, with tumor regions outlined in red and stromal regions in yellow. i, j) Immunofluorescence staining of poorly differentiated (i) and well‐differentiated (j) penile squamous cell carcinoma using antibodies against panCK (green), CD4 (yellow), CD8 (red), CD68 (purple), and CD20 (white). Nuclei were counterstained with DAPI (blue). Images were captured using laser confocal microscopy to illustrate the infiltration of immune cells in penile squamous cell carcinoma with varying degrees of differentiation. Scale bars: 50 µm. k) Cell‐cell communication analysis between tumor cells as sender cells and immune cell subtypes as receiver cells. The left panel shows a dot plot illustrating the expression patterns of ligands in tumor cells. The heatmap analyzes the regulatory potential of prioritized ligands from tumor cells and predicted target genes in immune cell subtypes. The bottom panel shows a dot plot analyzing the expression levels of target genes in immune cell subtypes.

To explore the regulatory mechanisms of the tumor immune microenvironment (TIME) during the progression of different tumor cell subtypes, we first categorized the distribution of various cell types surrounding each tumor subtype. Our analysis revealed a pronounced presence of immune cells surrounding Tum_1 tumor cells, whereas sensory neurons were predominantly found near Tum_2 cells and melanocytes near Tum_3 cells (**Figure** **5**a). This observation aligns with our spatial transcriptomics data and supports previous studies suggesting that tumor regions closer to the stroma and normal tissues exhibit higher levels of immune cell infiltration (Figure 1e). To gain further insights into the immune regulation within the TIME, we classified immune cells into two major groups—myeloid and lymphoid—and identified 13 distinct immune cell subtypes (Figure 5b). To assess cell interactions in greater detail, we performed cell‐cell interaction analysis in the region within a 100 µm radius around Tum_1 cells. Our observations revealed that APOE^+^ macrophages and cytotoxic T lymphocytes (CTLs) exhibited enhanced interactions with all three tumor subtypes, thereby highlighting the significant spatial interplay between immune and tumor cell subtypes (Figure 5c,d). Interestingly, when comparing interaction numbers within 200 µm to those within 100 µm radius, we found substantial increases in the interactions between SDC1^+^ plasma B cells and tumor cells, and between classical plasma B cells and tumor cells (Figure 5e). The intensity of these interactions appeared to be modulated by varying concentrations of immune cell subtypes surrounding the Tum_1 cells (Figure 5f). For example, APOE^+^ macrophages declined, while SDC1^+^ plasma B cells increased with greater distance from Tum_1 cells (Figure 5f,g). This shift in immune cell distribution was particularly noticeable at the invasive front of the tumor, where gathered basal stem‐like cells were found, whereas the outer lateral regions, associated with keratinizing tumor cells, exhibited reduced immune cell infiltration (Figure 5h).

To further explore the relationship between immune cell infiltration and tumor differentiation, we conducted immunofluorescence staining on pathological tissues from PSCC samples with varying degrees of differentiation. Poorly differentiated tumor regions exhibited significantly higher infiltration of T cells, B cells, and macrophages compared to more differentiated areas. We also validated the phenotype of Tum_1 and its associated TME in these samples, which provided further evidence supporting the existence of Tum_1 and its interactions with the TME (Figure 5i,j; Figure S4f (Supporting Information) and Table S3, Supporting Information). By analyzing the ligands corresponding to chemokine target genes expressed in immune cells, we found that the chemotactic response of immune cells was mediated by the expression of NRG1 and HMGB1 ligands on Tum_1 tumor cells, which interacted with the CXCL1 target on APOE^+^ macrophages (Figure 5k). This suggests that Tum_1 cells may actively recruit and modulate APOE^+^ macrophages through NRG1/HMGB1‐CXCL1 interaction. To further elucidate the interactions between APOE^+^ macrophages and the Tum_1 subtype, we designated immune cells as the signal‐emitting cells and Tum_1 cells as the signal‐receiving cells. Our analysis revealed that APOE^+^ macrophages acted on Tum_1 cells through CXCL1 and TNFSF10, inducing the expression of CXCL8 and MMP9 in Tum_1 (Figure S4g, Supporting Information). These findings emphasize the importance of APOE^+^ macrophages in fostering an environment that supports the aggressiveness of Tum_1 cells. The invasiveness supportive immune environment was also observed in ESCC cohort from the TCGA database by examining the correlation of Treg, exhausted T cells, and M2 macrophage gene signatures, with Tum_1 gene signatures (Figure S4h, Supporting Information).

### 2.6 HPV‐Positive Basal Stem‐Like PSCC is Characterized by Pronounced Immune Evasion and Suppression

**Figure 6:**
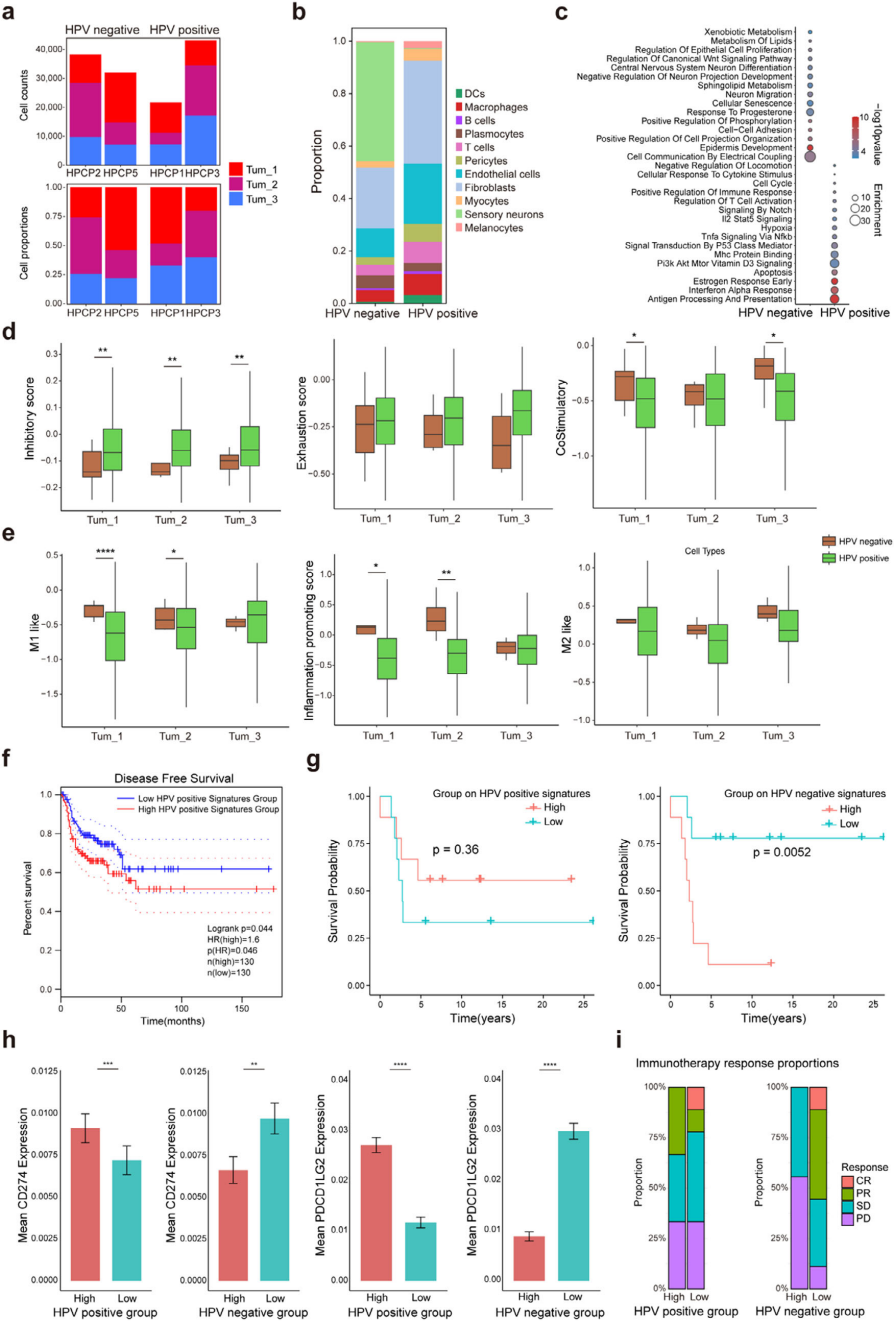
HPV infection regulates tumor immune microenvironment, affects prognosis, and immunotherapy efficacy. a) Stacked bar plot showing the Tum_1, Tum_2, Tum_3 subtype proportion and number in Stereo‐seq data, separated by HPV‐positive samples and HPV‐negative samples. b) Stacked bar plot representing the distribution of various cell types within HPV‐positive and HPV‐negative samples as derived from snRNA‐seq data. c) Dot plot illustrating the comparative analysis of enriched pathways between HPV‐positive and HPV‐negative tumors from snRNA‐seq data. The plot uses the ‐log10(p‐value) to signify the statistical significance of each pathway and enrichment score to quantify the extent of pathway enrichment. d) Box plots representing functional gene set scores of T cells within 30 um radius surrounding tumor subtype 1, 2, and 3 in the Stereo‐seq data, separated by HPV‐positive samples and HPV‐negative samples, statistically tested using the two‐sample t‐test. e) Box plots representing functional gene set scores of macrophages within 30 um radius surrounding tumor subtypes 1, 2, and 3 in the Stereo‐seq data, separated by HPV‐positive samples and HPV‐negative samples, statistically tested using the two‐sample t‐test. f) Disease‐free survival analysis assessing disease‐free intervals, contrasting groups characterized by high and low expression levels of specific marker gene set associated with HPV‐positive Tum_1, statistically tested using the Mantel‐Cox log‐rank test. g) Kaplan‐Meier survival curves delineate the disparities of survival probabilities in GSE93157 cohorts which received anti‐PD‐1 immunotherapy, separated by elevated and reduced expression of marker gene panel specific to HPV‐positive Tum_1 and, in parallel, HPV‐negative Tum_1, statistically tested using the Mantel‐Cox log‐rank test. h) Box plots showing gene expression levels of CD274 and PDCD1LG2 across different groups, specifically those with high and low expression of the marker gene set for HPV‐positive Tum_1 and HPV‐negative Tum_1. i) Stacked bar plot showing the outcomes of anti‐PD‐1 immunotherapy in patients with high and low expression of the marker gene set of HPV‐positive Tum_1 and HPV‐negative Tum_1, respectively. CR: complete response; PR: partial response; SD: stable disease; PD: progressive disease. Statistical significance is indicated by *p < 0.05, **p < 0.01, ***p < 0.001, ****p < 0.0001.

We aimed to further investigate the impact of HPV infection on tumor development. In terms of tumor cell subtypes, HPV status did not significantly alter the compositional proportions of tumor cell subtypes in PSCC at a statistical level (**Figure** **6**a).To assess whether HPV infection correlated with alterations in immune cell composition, we compared the immune cell distribution in both HPV‐positive and HPV‐negative samples, discovering that the former exhibited elevated proportions of immune cells, particularly T cells, macrophages, and plasma cells (Figure 6b). Moreover, functional comparative analyses between HPV‐positive and ‐negative tumor cells indicated that those infected by HPV demonstrated a stronger immune response, including enhanced antigen processing and presentation, as well as a more robust type I interferon response (Figure 6c; Figure S5a,b, Supporting Information).

Given that the entire section contained normal tissue, our focus for the research of the tumor immune microenvironment should be on the region surrounding the tumor cells. Therefore, in the subsequent analysis, we focused on three tumor subtypes, which were likely to provide more clinically relevant insights. An analysis of immunoinhibitory and exhaustion levels of T cells within a 30 mm radius from the three tumor subtypes showed that T cells adjacent to malignant cells from HPV‐positive tumors had significantly elevated inhibitory scores and reduced co‐stimulatory scores (Figure 6d). Scoring of macrophages revealed that in the HPV‐positive group, M1 phenotype and pro‐inflammatory scores of macrophages increased as they moved from basal stem‐like tumor cells toward keratinized tumor cells. Similar to T cells, macrophages adjacent to HPV‐positive Tum_1 cells displayed significantly reduced pro‐inflammatory scores, which indicated a shift towards a more immunosuppressive phenotype compared to HPV‐negative status (Figure 6e). Consequently, signatures for both HPV‐negative and ‐positive basal stem‐like (Tum_1) cells were computed, and TCGA data analysis indicated that head and neck squamous carcinoma (HNSCC) patients with a low expression of the HPV‐positive Tum_1 signature experienced prolonged recurrence‐free survival (RFS) (Figure 6f). Furthermore, survival analysis on a cohort treated with anti‐PD‐1 therapy demonstrated that HNSCC and LSCC patients with a heightened expression of the HPV‐positive Tum_1 signature displayed improved survival following treatment, although statistical significance was not achieved.^[^ ^35^ ^]^ Conversely, those with a high expression of the HPV‐negative Tum_1 signature encountered significantly poorer post‐treatment survival (Figure 6g). This finding aligned with the expression levels of PD‐L1 and PD‐L2 in the respective HPV‐positive and HPV‐negative Tum_1 cell groups in our data (Figure 6h). To further explore the role of HPV in PSCC, this finding was validated using mIF staining on tissue samples from our validation cohort (Figure S5c, Supporting Information). Regarding treatment response, partial response (PR) was noted among patients with a high HPV‐positive basal stem‐like signature, while those with a high HPV‐negative basal stem‐like signature consistently showed stable disease or disease progression (SD/PD) (Figure 6i). Consequently, we hypothesize that patients with characteristics of HPV‐positive basal stem‐like PSCC can derive greater benefit from PD‐1 blockade therapy.

## 3 Discussion

Our study presents a comprehensive single‐cell and spatially resolved transcriptomic atlas of Penile Squamous Cell Carcinoma (PSCC), providing unprecedented insights into the tumor's cellular and molecular architecture.^[^ ^1^, ^36^ ^]^ By integrating single‐nucleus RNA sequencing (snRNA‐seq) with high‐resolution spatial transcriptomics, we systematically mapped the differentiation trajectories of tumor epithelial cells and characterized key features of the tumor microenvironment (TME). Our findings uncover potential therapeutic targets such as LAMC2, which promotes tumor invasiveness. Additionally, we found that HPV‐positive PSCC tumors exhibit a distinct immunosuppressive microenvironment, characterized by elevated inhibitory scores in T cells and reduced pro‐inflammatory scores in macrophages. These findings suggest that HPV‐positive PSCC may respond better to immune checkpoint inhibitors, such as PD‐1 blockade therapy, due to the unique immune landscape. Overall, our results provide a foundational framework to enhance our understanding of PSCC pathogenesis and guide the development of future therapeutic strategies, including personalized immunotherapies for HPV‐positive patients.

While previous studies on PSCC have utilized bulk transcriptomics, single‐cell RNA‐seq, or conventional immunohistochemistry to explore its molecular landscape, these approaches lack the spatial cellular resolution needed to disentangle the complexity of the tumor and its microenvironment.^[^ ^1^, ^22^, ^37^, ^38^ ^]^ Our study represents the first high‐resolution spatial transcriptomic investigation of PSCC, bridging this critical gap by revealing tumor evolution at the single‐cell level within its native spatial context. This method allows us to pinpoint distinct spatial cellular subpopulations, trace developmental trajectories, and delineate spatial interactions between tumor and stromal cells. Compared to prior PSCC studies that primarily focused on HPV‐related transcriptional changes or limited immune profiling, our approach provides a more nuanced understanding of tumor progression and immune modulation, positioning it as a significant leap forward in PSCC research.^[^ ^39^ ^]^

With this advanced approach, we demonstrate that PSCC epithelial cells undergo a tumorigenesis process independent of HPV infection, characterized by a conserved three‐tiered developmental structure. This mirrors observations in other squamous cell carcinomas, such as ESCC and cervical squamous cell carcinoma, where basal, intermediate, and keratinocyte layers define tumor differentiation trajectories.^[^ ^15^, ^31^, ^40^, ^41^ ^]^ Notably, the Tum_1 spatial subtype identified in our study exhibits basal‐like characteristics with stemness features, suggesting its role as an early‐stage precursor population during tumorigenesis. While HPV infection is known to contribute to immune modulation and can influence the overall tumor microenvironment, our results indicate that the fundamental differentiation process of PSCC tumor cells remains consistent regardless of HPV status. This intrinsic differentiation pattern suggests that targeting the early‐stage precursor cells, such as Tum_1, could be a promising therapeutic strategy for PSCC, potentially applicable across both HPV‐positive and HPV‐negative cases. Understanding the molecular and cellular mechanisms underlying this conserved differentiation trajectory will provide valuable insights into the early events of PSCC tumorigenesis and pave the way for the development of more effective, targeted therapies.

Our study identified *LAMC2* as a key molecular driver in PSCC, particularly in the Tum_1 spatial subtype, where it mediates tumor progression through laminin‐integrin binding. The interaction of *LAMC2* with ITGA6/ITGB4 activates downstream signaling pathways that enhance tumor invasiveness, a mechanism also observed in other malignancies such as pancreatic ductal adenocarcinoma, colorectal cancer, and lung adenocarcinoma.^[^ ^42^, ^43^, ^44^ ^]^ *LAMC2*’s elevated expression in PSCC and its association with basal‐like, stemness‐enriched subpopulations underscore its potential as a therapeutic target. Experimental evidence in other cancers has demonstrated that targeting laminin‐integrin interactions can reduce tumor cell invasiveness and metastasis, supporting the feasibility of translating *LAMC2*‐based therapeutic strategies into clinical practice.^[^ ^45^ ^]^ In our study, we preliminarily validated the critical role of *LAMC2* in promoting tumor invasiveness in PSCC through functional experiments. At the cellular level, *LAMC2* knockdown significantly inhibited tumor cell migration and invasion capabilities, while also reducing the expression of key molecules associated with integrin signaling pathways. While these functional experiments highlight the importance of *LAMC2* in PSCC, further investigations are needed to determine its applicability in different tumor subtypes and clinical contexts. For example, it is essential to explore whether *LAMC2* specifically influences the invasive phenotype of Tum_1 tumor subtype and whether it can synergize with existing therapeutic strategies, such as immune checkpoint inhibitors, to enhance therapeutic outcomes. Additionally, therapeutic strategies targeting *LAMC2*, such as specific inhibitors or antibody‐based therapies, must be validated in larger preclinical and clinical trials to assess their safety and efficacy.

Given our findings, HPV‐positive basal stem‐like PSCC may exhibit enhanced responses to PD‐1 blockade therapy. Stratifying patients based on this signature could help identify those most likely to benefit from immune checkpoint inhibitors, enabling more precise and effective interventions. To test the therapeutic potential of targeting the Tum_1 subpopulation or its associated pathways, preclinical validation using patient‐derived organoids and xenograft models is needed to assess the role of Tum_1‐related pathways, such as *LAMC2*, in tumor progression, as well as the efficacy and safety of targeted therapies. For clinical validation, patients with high Tum_1 signature expression should be prioritized in trials. In HPV‐negative cases, therapies targeting *LAMC2* may be promising, while in HPV‐positive patients, combining PD‐1 blockade with Tum_1‐targeted approaches may offer enhanced benefit.

However, our study has limitations. The relatively small sample size may reduce statistical power and limit generalizability. Moreover, the cohort's restricted age range (mostly 40 and 70 s) and disease stages (mainly T1 and T3) may introduce bias. Future studies should include larger and more diverse populations to strengthen the robustness of our conclusions. Functional validation of Tum_1 markers, particularly *LAMC2*, in preclinical models will be critical to confirm their role in tumor progression and treatment response. Longitudinal studies across HPV‐positive and HPV‐negative cohorts will further clarify HPV status–associated therapeutic effects and support the development of personalized treatment strategies.

In conclusion, our study provides a comprehensive spatial map of PSCC, revealing the molecular and cellular mechanisms underlying its pathogenesis. The identification of *LAMC2* as a key driver of tumor invasiveness highlights its potential as a therapeutic target in PSCC. In contrast, HPV‐positive tumors, with their suppressive immune landscape, represent ideal candidates for immunotherapy, including checkpoint inhibitors and therapeutic vaccines. The differential characteristics of HPV‐positive and HPV‐negative PSCC underscore the need for stratified treatment approaches tailored to their unique molecular and immune profiles. Future research should focus on validating *LAMC2*‐targeted therapies in preclinical and clinical settings and exploring the combination of immunotherapy with HPV‐directed vaccines. Additionally, integrating multi‐omics data and longitudinal sampling could provide deeper insights into the dynamic evolution of PSCC and its response to therapy. By addressing these challenges, our findings lay the groundwork for the development of more effective, personalized therapeutic strategies for PSCC, ultimately improving patient outcomes in this aggressive malignancy.

## 4 Experimental Section

### Clinical Specimens and Data for Spatial Stereo‐seq, snRNA‐seq, and Validation Cohorts

All samples were derived from patients with penile cancer who underwent penectomy at Harbin Medical University. Six penile cancer samples were included in this study, of which two were HPV‐positive and four were HPV‐negative. The study protocol was approved by the Ethics Committee of Harbin Medical University Cancer Hospital (approval number: KY2024‐04). All subjects signed a written informed consent form, explicitly agreeing to use anonymous patient data for academic publication. Patient samples were designated as “HPCP”, representing “Human Penile Cancer Patients”, and the corresponding clinical information is provided in Table S1, Supporting Information. The freshly collected samples were immediately immersed in RNA‐safer protective solution and then embedded in OCT for preservation. Consecutive sections were cut for each sample, and the sections were stained with Hematoxylin and Eosin (H&E). Pathologists identified and marked each area, including tumor areas, normal epithelial tissue, and stromal areas. All samples underwent single‐cell sequencing, and four samples with clear anatomical and tissue morphology were further subjected to Stereo‐seq spatial transcriptome sequencing.

For the validation cohort, samples of thirty‐three penile cancer patients were included. The study protocol was approved by the Ethics Committee of Harbin Medical University Cancer Hospital (approval number: KY2024‐21). All subjects signed a written informed consent form, explicitly agreeing to use anonymous patient data for academic publication. Patient samples were designated as “HPCPV”, representing “Human Penile Cancer Patients for Validation”, and the corresponding clinical information is provided in Table S3, Supporting Information. Formalin‐fixed paraffin‐embedded (FFPE) samples were subjected to multiplexed immunofluorescence (mIF) and immunohistochemistry (IHC) analyses.

### Preparation of Single‐Nucleus Suspension

To obtain nuclear suspension for single‐nucleus RNA sequencing, all six samples were prepared according to well‐defined protocols.^[^ ^46^ ^]^ Briefly, during the grinding step, frozen penile tissue sections were placed in a Dounce homogenizer containing 2 mL of pre‐chilled homogenization buffer. Fifteen homogenizations were performed using a type A pestle, followed by another 15 homogenizations using a type B pestle. Afterwards, an additional 2 mL of homogenization buffer was added to the homogenate, and the resulting homogenate was filtered through 30 µm MACS Smart Filter (Miltenyi Biotech, #130‐110‐915) into a 15 mL conical tube. The filtered suspension was centrifuged at 500 g for 5 min at 4 °C to pellet the nuclei. The pellet obtained was resuspended in 1.5 mL of blocking buffer, and the centrifugation step was repeated under the same conditions (4 °C, 500 g, 5 min) to obtain a nuclear pellet again. Finally, the isolated nuclei were resuspended in cell resuspension buffer and used for subsequent single‐nucleus RNA sequencing library construction.

### SnRNA‐seq Library Construction and Sequencing

Sequencing libraries were constructed using the DNBelab C Series High‐Throughput Single‐Cell RNA Library Preparation Kit (MGI, No. 940‐000047‐00) following the standard protocol provided by the manufacturer. Specifically, single‐nuclear suspensions were used in a series of key steps, including droplet generation, emulsion breaking, bead collection, reverse transcription, second‐strand synthesis, cDNA amplification, and amplification of droplet index products to construct barcoded sequencing libraries. Subsequently, the library was quantitatively detected using the Qubit ssDNA Assay Kit (Thermo Fisher Scientific, No. Q10212). Library sequencing was completed by the Shenzhen National GeneBank of China, and data were generated using the ultra‐high‐throughput DIPSEQ T1 or DIPSEQ T10 sequencing platform.

### SnRNA‐seq Data Processing

Upon acquisition of the FASTQ file for each sample, alignment was conducted using STAR‐v2.7.1a to the GRCh38 genome reference (Ensembl 98).^[^ ^47^ ^]^ Subsequently, PISA v0.7‐24^[^ ^48^ ^]^ facilitated the modification of the Mapping Quality (MAPQ) score, the filtration of alignments, the annotation of genes, and the correction of the unique molecular identifiers (UMI). The resultant data from this process was a UMI count matrix per sample, which underwent transformation into a Seurat object via the Seurat package (v.4.3.0)^[^ ^49^ ^]^ in R (v4.2.0). The applied quality filters encompassed criteria designed to exclude low‐quality barcodes, specifically: an inadequate UMI count (<1000); a suboptimal number of genes expressed by cells (<500); an excessive number of genes expressed by cells (>8000); overrepresentation of mitochondrial gene expression in total transcript counts (>10%); a high percentage indicative of potential doublets (>0.05%); and a possible indication of dead cells. The final step involved the amalgamation of datasets from all samples, resulting in a collection of 96904 single cells. The Scanpy package (v1.10.0)^[^ ^50^ ^]^ within the Python environment (v3.9.16) was utilized for data manipulation. The “normalize_total” and “log1p” functions from the preprocessing module were employed to perform logarithmic normalization of the UMI count matrix. Subsequent to the identification of the top 2000 highly variable genes via the “highly_variable_genes” function, principal component analysis (PCA) was conducted to diminish the dimensionality of the snRNA‐Seq dataset. To address batch effects, the “harmony_integrate” function from the scanpy^[^ ^51^ ^]^ was applied, ensuring harmonized data integration across batches. The “neighbors” function was executed to define neighbors, followed by the application of the Leiden algorithm for cellular clustering. For visualization and further analysis, Uniform Manifold Approximation and Projection (UMAP) was implemented to map the high‐dimensional snRNA‐Seq data onto a two‐dimensional plane.

### SnRNA‐seq Data Cell Type Annotation

The cellular identities of individual cells were inferred by leveraging the spatial distribution of clusters and the expression profiles of cell‐type‐specific marker genes visualized on UMAP projections. Subsequent clustering analysis enabled the classification of cells into distinct cell types. Epithelial cells were identified with reference to established cell types, including B cells, T cells, macrophages, and endothelial cells. To distinguish normal epithelial cells—characterized by the absence of copy number variation (CNV) events—from neoplastic epithelial cells, the R package inferCNV (v1.12.0; github.com/broadinstitute/infercnv) was employed. During the execution of the “infercnv::run” function, key parameters were configured as follows: “cutoff” was assigned a value of 0.1, “sd_amplifier” was set to 1.5 to enhance CNV signal sensitivity, and a Hidden Markov Model (HMM) was implemented for robust CNV state inference.

### Tissue Sectioning, Fixation, Staining, and Imaging

Tumor and adjacent normal tissues were processed for sectioning, fixation, staining, and imaging, with necrotic regions and areas proximal to major blood vessels systematically excluded. Tumor samples embedded in optimal cutting temperature (OCT) compound were cryosectioned into 10 µm thick slices using a Leika CM1950 cryogenic slicer. The resulting sections were carefully transferred onto the surface of Stereo‐seq chips and fixed using a two‐step methanol‐based protocol: incubation at 37 °C for 3 min followed by −20 °C for 30 min. Subsequently, the sections were stained with a nucleic acid dye (Thermo Fisher, Q10212) to facilitate the visualization of single‐stranded DNA (ssDNA). Imaging was performed directly on the chips using a Motic PA53 FS6 microscope, to enable high‐resolution spatial profiling of the samples.

### Stereo‐seq Library Construction and Sequencing

The Stereo‐seq libraries were prepared using the STOmics Gene Expression kit S1 (BGI, 1000028493) following the standard protocol.^[^ ^18^ ^]^ The sequencing process was carried out on the DNBSEQ T1 sequencing platform (China National GeneBank), MGISEQ‐2000, or DNBSEQ‐T7 sequencing platform (BGI‐research, Beijing) in paired‐end mode. Each sequencing read consisted of a 50 bp Read 1 and a 100 bp Read 2.

### Stereo‐seq Data Processing

The FASTQ files were aligned to the reference genome (GRCh38) using the STAR algorithm^81. ^Coordinate identities (CIDs) were determined by aligning them with the coordinates of the in situ‐captured chip. One base mismatch was allowed during the alignment process to tolerate potential sequencing errors and errors introduced by PCR. After the alignment was completed, reads with a MAPQ score higher than 10 were filtered out, quantified, and attributed to the corresponding gene. Subsequently, unique molecular identifiers (UMIs) with the identical CID and gene locus were integrated to generate an expression profile matrix containing CID information. The final data also includes an additional file named cell mask image, which records the spatial location information corresponding to each CID sequence. For a detailed workflow of this method, please refer to https://github.com/STOmics/SAW.^[^ ^52^ ^]^

To achieve cell segmentation, the slices were first stained with nucleic acid, and the stained images were projected onto the Stereo‐seq chip. Subsequently, the nucleic acid stained images of each field of view were automatically spliced, and the effects of field overlap and chip grid lines were considered through meticulous manual adjustments. When generating the cell expression matrix, the expression data on the chip were rendered as grayscale images, and their grayscale intensity was positively correlated with the UMI count at each spot. After the chip grid lines were superimposed on the grayscale image, they were manually aligned with the nucleic acid stained image, and finally, the registered stained image was output as the registered cell‐mask image. These processes were implemented using the StereoCell software.^[^ ^53^ ^]^

The Stereopy algorithm utilizes the “Single Strand DNA tissue Cut” and “cell_seg_v3” functions to perform tissue segmentation and cell segmentation on the registered images, respectively. It was important to note that the automated processing may incorrectly perform cell segmentation operations in the background area of the image. Therefore, in order to reduce the impact of background interference, a tissue segmentation filtering step needs to be introduced to optimize the accuracy of cell segmentation.

In the process of integrating tissue images with gene expression matrices, Stereopy uses the “cell_correct” function to correct cell data using the Gaussian Mixture Model (GMM) method. After correction, a single‐cell expression matrix with spatial resolution was generated, which contains cell label, gene expression data, and centroid position information, and was encapsulated in the Anndata data structure.

### Spatial Cell Type Annotation and Domain Partitioning

Initially, genes expressed in fewer than 10 cells were filtered out using the “scanpy.pp.filter_genes function. The gene expression matrix was subsequently normalized and log‐transformed via the ‘scanpy.pp.normalize_total” and “scanpy.pp.log1p” functions, respectively. Cell annotations were assigned utilizing the Spoint module from the SPACEL package (v1.1.6),^[^ ^25^ ^]^ which computed deconvolution scores for each cell based on Stereo‐seq data. These scores were matched to cell type labels derived from snRNA‐seq data, and the cell type with the highest score was designated for each cell.

### Epithelial Subtype Characterization

To identify epithelial cell subtypes of snRNA‐seq data, batch effects were corrected using the “harmony_integrate” function from the scanpy.external package. The Leiden algorithm was applied for cellular clustering. Differential gene expression analysis was conducted using the Seurat package to identify epithelial subtype characteristics. The “FindAllMarkers” function was applied to compare the gene expression profiles of the three epithelial layers in normal penile epithelium (BS, BK, DK) with their counterparts in penile tumor epithelium. Significantly differentially expressed genes (DEGs) were identified based on the following criteria: adjusted *p*‐value < 0.05, log2 fold‐change > 0.5, and proportion of expressing cells in the cluster (≥ 0.3). To further differentiate tumor from normal epithelial gene signatures, overlapping genes between tumor and normal epithelium (specific to each layer) were removed, and unique gene sets were determined as their feature gene set.

Once DEGs were identified for each cluster, functional enrichment analysis was carried out using the Metascape^[^ ^54^ ^]^ platform with default settings. This analysis integrated various pathway categories, including Gene Ontology (GO) molecular functions, GO biological processes, canonical pathways, Reactome gene sets, KEGG pathways, BioCarta gene sets, and hallmark gene sets.

### Pseudotime Trajectory Inference

To characterize the potential phenotypic transition of epithelial cells, pseudotime trajectory analysis was performed using Monocle2 (v2.32.0).^[^ ^55^ ^]^ Epithelial cells were analyzed by selecting the top 500 DEGs across groups, with selection criteria set to log2 fold‐change > 0.5 and adjusted *p*‐value < 0.001. The trajectory was constructed using the “DDRTrees” method implemented in the “reduceDimension” function. The resulting trajectory path was visualized using the “plot_complex_cell_trajectory” function.

For the expression of individual genes along the pseudotime, the expression values were log‐transformed (base 2) with an offset of 1 added to avoid undefined values for zero expression. Additionally, to evaluate the expression patterns of gene sets along the pseudotime trajectory, the average expression value for each gene set was calculated across the dataset. These average values were visualized in the pseudotime trajectory using the “plot_cell_trajectory” function, providing insights into the coordinated expression dynamics of specific gene sets during the phenotypic transition process.

To visualize gene expression patterns and identify “cluster‐specific” functional features, the ClusterGV is utilized package (v0.1.1)^[^ ^56^ ^]^ to analyze the time series gene expression data. Marker genes for each cluster were identified using the “FindAllMarkers” function in the Seurat package, and the top 20 genes for each cluster were selected based on the average log2 fold change value. Other biologically significant genes were manually merged into the marker set to ensure comprehensive coverage. Enrichment analysis was performed on each cluster using the “enrichCluster” function that was seamlessly integrated with the clusterProfiler package (v4.12.6),^[^ ^57^ ^]^ using org.Hs.eg.db as the database and a *p*‐value threshold of 0.05. Functional enrichment analysis was performed on the top cluster markers, and the top five enriched pathways were identified. The visualization of cluster gene expression patterns included line graphs, heat maps, and combined cluster heat maps with functional annotations generated by the “visCluster” function. The “plot_genes_branched_heatmap2” function was used to perform pseudo‐temporal branch analysis, determine the distribution of differential genes on different branches, and visualize them using the “visCluster” function.

### Gene Expression Visualization on Spatial Plot

For visualizing gene expression within a spatial context, a convolution of the spatial expression profile matrix was performed to generate an N × N binned expression profile matrix, denoted as binN (with N set to 50). Preprocessing of the bin50 data mirrored that of snRNA‐seq data, except for the cell clustering step. The gene expression data were normalized and scaled before being visualized. Spatial gene expression plots were generated using the “SpatialFeaturePlot” function from the Seurat package, with the stroke parameter set to 0 to produce a cleaner visual representation.

### Transcription Factor Analysis

The analysis of transcription factors (TFs) was conducted using pySCENIC (v0.12.1).^[^ ^58^ ^]^ The required TF list and database files were sourced from the Aerts Lab's resource page. The SCENIC pipeline was followed, which consisted of three key stages: first, GENIE3 was used to build a co‐expression network linking TFs with their target genes; next, RcisTarget was applied to validate potential regulatory interactions between TFs and genes through DNA motif analysis; and finally, the activity of regulons in each cell was quantified by calculating the Area Under the Curve (AUC) with AUCell. To pinpoint TFs specific to certain cell types, the regulatory similarity score was calculated across different cell subtypes using the “calcRSS” function. The visualization of these cell type‐specific TFs was facilitated by the “plotRSS” function, with the “zThreshold” parameter set to 1 and the “thr” parameter to 0.1.

### Cell Aggregation

To enhance mRNA capture efficiency at individual DNA nanoball (DNB) sites with a subcellular resolution of 500 nm, gene counts from adjacent cells of the same cell type were aggregated. This process involved combining gene counts from a central cell and its 30 nearest neighbors. Spatial clustering was performed using the Bisecting K‐Means algorithm from the Python scikit‐learn package (v1.1.3),^[^ ^59^ ^]^ with the “bisecting_strategy” parameter set to largest_cluster. The number of clusters was determined by dividing the total number of cells by 30. Following this aggregation, the gene counts were normalized, resulting in a dataset with lower spatial resolution but improved mRNA capture efficiency. These aggregated clusters were subsequently analyzed for differential gene expression, functional enrichment, and cell‐cell communication.

### Cell Neighboring Analysis

Spatial proximity between cells was quantified using the Squidpy package (v1.2.3).^[^ ^60^ ^]^ The “spatial_neighbors” function was employed to identify neighboring cells within radii of 30, 50, 100, 200, 300, and 400 µm from each cell centroid, with the “coord_type” parameter set to generic. The final proportions were calculated as the ratio of neighbors of different cell types to the total number of neighbors. The analysis concluded with an evaluation of the relative proportions of various immune cell subtypes in proximity to tumor cells.

### Cell Communication Analysis

Cell chat analysis was conducted by the CellChat package (v1.6.1).^[^ ^61^ ^]^ To explore intercellular communication at the ligand‐receptor level, A CellChat object was first constructed using the scRNA‐seq expression matrix. Overexpressed ligands and receptors were identified using the “identifyOverExpressedGenes” function, followed by the inference of significantly overexpressed ligand‐receptor interactions with the “identifyOverExpressedInteractions” function. These interactions were subsequently projected onto the protein‐protein interaction (PPI) network to integrate known ligand‐receptor pairs into a comprehensive signaling framework. Communication probabilities between cells were computed using “computeCommunProb” function, while pathway‐level communication probabilities were inferred via “computeCommunProbPathway” function. The aggregated communication networks were generated and summarized using “aggregateNet” function. The ligand‐receptor network between specific cell types was visualized by “netVisual_bubble” function. To facilitate comparative analyses of cell‐cell interactions between two CellChat objects, the “mergeCellChat” function was applied to integrate multiple objects. “netVisual_heatmap” function was applied to display the interaction differences among cell types between CellChat objects.

NicheNet analysis was conducted by the NicheNet package (v2.1.3).^[^ ^62^ ^]^ The NicheNet approach enables the prediction of ligand‐to‐target signaling relationships by leveraging a comprehensive regulatory network that integrates ligand‐receptor interactions, ligand‐target links, and gene regulatory networks. Key database resources, including ligand‐receptor networks, ligand‐target matrices, and regulatory interactions, were downloaded from https://zenodo.org/record/7074291/files. Differentially expressed ligands were identified in sender cells based on the following thresholds: log2 fold‐change > 0.5, adjusted *p*‐value < 0.05, and pct > 0.1. Ligand activity was predicted using the “predict_ligand_activities” function, which ranked candidate ligands based on their capacity to activate downstream targets. To establish ligand‐target relationships, the “get_weighted_ligand_target_links” function was employed, exporting weighted links between ligands and their predicted target genes. Potential ligand targets were filtered using a defined weight threshold of 0.6, as specified in the “prepare_ligand_target_visualization” function, to facilitate the identification of high‐confidence target genes.

### Gene Set Score Calculation

The “AddModuleScore” function from the Seurat package was employed to assess the activity of specific gene sets at the single‐cell level. This function computes module scores by first calculating the average expression of genes within a predefined gene set and then subtracting the average expression of a randomly selected control gene set. The control genes were chosen from expression bins based on their average expression levels to account for technical variability and biases. Following this, the “ctrl” parameter was set to 100, directing the algorithm to randomly sample 100 control genes for each expression bin to generate a robust background reference. The resulting module scores, representing the relative activity of each gene set, were subsequently added to the Seurat object's metadata. These scores were utilized for downstream analyses, including visualization and statistical assessment.

### Multiplexed Immunofluorescence (MIF) Analysis

FFPE (formalin‐fixed, paraffin‐embedded) tissue blocks from marginal regions (2 × 2 × 1 cm, centered within a 2 cm radius around the tumor boundary) were sectioned into 4 µm‐thick slices. Multiplex immunofluorescence (mIF) staining was performed using the PANO 6‐color IF kit (Panovue, Beijing, China). FFPE sections were deparaffinized with xylene, rehydrated through a graded ethanol series, and subjected to antigen retrieval in Tris‐EDTA buffer (pH 8.0) using microwave heating, followed by cooling to room temperature. Endogenous antigen activity was blocked by incubating slides with 1% bovine serum albumin (BSA) at room temperature for 30 min. Primary and secondary antibody incubations were performed at room temperature for 1 hour and 30 min, respectively, with fluorescent tyramide signal amplification (TSA) reagents (PPD 520, PPD 570, PPD 620, PPD 650, and PPD 780; Panovue) applied sequentially for 10 minutes at each step. After counterstaining with DAPI for 5 min, the slides were washed with TBST buffer between steps, mounted using anti‐fade medium (P36971, ThermoFisher Scientific), and stored at 4 °C. Imaging was performed with a Zeiss confocal microscope, and multispectral image analysis was conducted using InForm software (PerkinElmer), including autofluorescence removal and quantitative evaluation of marker expression. The primary antibodies are shown in Table S4, Supporting Information.

### Immunohistochemistry Analysis

The tissue slices were prepared as mentioned in *MIF analysis*. The tissue on the slides underwent a meticulous dewaxing and hydration process, followed by blockade with 1% bovine serum albumin to ensure optimal conditions. Subsequently, the P40 and LAMC2 antibody (1:500) were incubated at 4 °C overnight, while the goat‐anti‐rabbit antibody (1:1000) was incubated at 37 °C for 1 h. The utilization of diaminobenzidine (SignalStain DAB Substrate Kit, #8059, Cell Signaling Tech) yielded satisfactory staining outcomes. Finally, hematoxylin was employed for staining, and a series of meticulously selected reagents, including gradient alcohol and xylene, were used for dehydration. A neutral adhesive was applied for sealing, and the tissue was observed under a microscope (PrimovertHDcam, ZEISS, Germany). The primary antibodies are shown in Table S4, Supporting Information.

### Cell Culture and Transfection

Penile squamous cell carcinoma cell line Penl1 was obtained from the National Biomedical Experimental Cell Repository. Cells were cultured in Dulbecco's modified Eagle's medium (DMEM; Thermo Fisher Scientific, China) with 10% fetal bovine serum (FBS; Thermo Fisher Scientific, China) and kept in a 37 °C incubator supplied with 5% CO2. HMGA2 or LAMC2 cDNA sequences were cloned into the plasmid vector pcDNA3.1 (GenScript, Nanjing, China) to construct HMGA2 or LAMC2 overexpression plasmids. Four specific siRNAs targeting HMGA2 (siHMGA2#1, siHMGA2#2) and LAMC2 (siLAMC2#1, siLAMC2#2) were designed for gene silencing. Penl1 cells were transfected with either the HMGA2 or LAMC2 overexpression plasmids or the corresponding siRNAs using Lipofectamine 2000 reagent (Invitrogen, USA; catalog #11668019), following the manufacturer's protocol.

### Quantitative Real‐Time PCR (qRT‐PCR)

Total RNA was isolated from cells (1×10^6^) with a Total RNA extractor (Trizol) kit (B511311, Sangon, China), and then the concentrations of total RNA were determined. Subsequently, total RNA (1.5 µg) from each group was used to synthesize into cDNA using oligo (dT)15 and random primer, and then 1 µL of cDNA was subjected to qRT‐PCR analysis. The PCR primers of HMGA2, LAMC2 and GAPDH were as follows: HMGA2 forward: 5ʹ‐ACCCAGGGGAAGACCCAAA‐3ʹ, reverse: 5ʹ‐CCTCTTGGCCGTTTTTCTCCA‐3ʹ, LAMHMGAC2 forward: 5ʹ‐GACAAACTGGTAATGGATTCCGC‐3ʹ, reverse: 5ʹ‐TTCTCTGTGCCGGTAAAAGCC‐3ʹ, and GAPDH forward: 5ʹ‐GACCTGACCTGCCGTCTAG‐3ʹ, reverse: 5ʹ‐AGGAGTGGGTGTCGCTGT‐3ʹ. The iTaq Universal SYBR Green (1725124, Bio‐Rad) was used to perform RT‐qPCR analysis. The following reaction conditions: 94 °C for 5 min, followed by 40 cycles at 94 °C for 10 s, 60 °C for 20 s, and 72 °C for 30 s. The relative mRNA levels were normalized to GAPDH and analyzed using the 2^−ΔΔ CT^ method.

### In Vitro Cell Proliferation Assay

An equal number of cells (3000 cells per well) was placed in a 96‐well plate and kept in an incubator (37 °C, 5% CO2). After adding 10 µL CCK8 (Cell Counting Kit‐8, HY‐K0301, MCE) reagent into each well, cells were incubated 2 h in an incubator (37 °C, 5% CO2) and absorbance at 450 nm was measured to determine cell proliferation rates.

### Wound‐Healing Assay

The transfected Penl1 cells were seeded in 12‐well plates. After the cells were grown to 80% confluence, scratch wounds were generated by a 200 µL plastic pipette tip, which was recorded as 0 h. Cell migration was assessed by measuring the closure of the scratch area over time. Then, the scratch was imaged at 48 h. Cell migration was quantified based on scratch closure using image analysis.

### Transwell Invasion Assay

For the transwell invasion assay, a 12‐well plate transwell chamber system (Corning, USA) was used. In the upper chamber (Corning, USA), 2×10^4^ transfected Penl1 cells were suspended in 200 µL serum‐free medium in triplicates, while 800 µL 10% FBS medium was added in the lower chamber to stimulate cell invasion action. After 48 h of incubation, the cells in the upper chamber were wiped with a cotton swab identical to the colony, and the cells entering the other side of the membrane were fixed with 4% PFA for 20 min and stained with 0.1% crystal violet. The stained chambers were air‐dried, imaged using a light microscope, and the number of invaded cells was counted.

### Public Data Analysis

To evaluate the clinical relevance of identified gene sets, expression and survival analyses were performed using external datasets. Gene expression levels and survival associations were assessed in squamous cell carcinoma (SCC) datasets via the GEPIA2 platform,^[^ ^63^ ^]^ which integrates TCGA and GTEx data to reveal prognostic implications. Additionally, transcriptomic and clinical data from the GSE93157 cohort were analyzed, focusing on two SCC subtypes, including HNSCC and LSCC, to examine the relationships between expressions of gene sets and responses to anti‐PD‐1 immunotherapy. Survival analysis was performed to assess differences in survival outcomes between groups using the“survdiff” and“survfit” functions from the survival package (*source: github.com/therneau/survival*). The survival curves were visualized using the“ggsurvplot” function from the survminer package (*source: github.com/kassambara/survminer*), providing insights into the association between gene expression scores and clinical outcomes.

### Statistical Analysis and Data Visualization

The ggplot2 package was utilized to generate boxplots for visualizing the distribution of data across different scoring groups and assessing the differences in their central tendencies. To determine whether the observed differences were statistically significant, the Wilcoxon test and two‐sample t‐test were conducted by the rstatix package (v0.7.2). Survival curves were compared using the Mantel‐Cox log‐rank test. The combined workflows for graph creation and statistical testing were executed in the R programming environment (v4.2.0) and Python (v3.9.16), leveraging their respective analytical and visualization capabilities. For the Penl1 cell experiment, Prism 8 software (version 8.2.1) was used for statistical analysis. Data were expressed as the mean ± standard error of the mean (SEM), and each experiment was independently conducted at least three times. Two‐tailed unpaired Student's t‐test was utilized for comparing data between two groups. The sample size (n) was shown in the corresponding figure legends. *p* value less than 0.05 was considered statistically significant. ****p < 0.0001, ***p < 0.001, **p < 0.01, *p < 0.05, n.s. means no significance.

## Conflict of Interest

The authors declare no conflict of interest.

## Author Contributions

H.S., Z.T., G.X., Y.L., Y.Z., and F.F. contributed equally to this work. All authors contributed significantly to the study's design or execution, including data acquisition, data processing, clinical sample management, or paper revision. All authors approved the paper. Each author is accountable for their individual contributions and has ensured the coherence, accuracy, and integrity of all aspects of the work. H.S., Z.T., G.X., Y.L., Y.Z., F.F., and D.W. conceived and designed the study. W.X., Z.T., W.D., Z.W., and Z.D. supervised the project. D.W., G.X., F.F., Z.Y., Q.Z., and F.W. conducted single‐cell sequencing, genetic data, and microenvironment analysis. H.S., Y.L., Y.Z., H.M. H.L., C.L., L.T., Y.X., D.D., T.X., J.L., and Y.C. collected clinical samples and performed data analyses. Q.S., Z.Z., Z.Y., Z.L., J.W., P.Z., P.D., Q.Y., X.H., and W.L. performed validation experiments. Z.T., D.W., and H.S. drafted the manuscript. H.S., Z.T., G.X., W.L. Y.Z., P.G., D.H., L.W., R.N., G.L. H.S., and D.W. contributed to research and manuscript revisions.

## Supporting information



Supporting Information

Supplementary Table 1

Supplementary Table 2

Supplementary Table 3

Supplementary Table 4

## Data Availability

The raw data of snRNA‐seq and Stereo‐seq data in this study are deposited into the GSA‐Human database of the National Genomics Data Center under accession code HRA009806, and the DAC (Data Access Committee) authorization is mandated by policy constraints. The data have also been deposited into the China National GeneBank Database (CNGB) with accession number CNP0006673. All the codes and scripts used to generate the results in this study are available on https://github.com/guixiang228/. The pre‐processing and intermediate processing scripts and tools could be found on DCS Cloud (https://cloud.stomics.tech/).
