# Supplementary material for: Single‐Cell and Spatial Transcriptomic Profiling of Penile Squamous Cell Carcinoma Reveals Dynamics of Tumor Differentiation and Immune Microenvironment
Source: Adv Sci (Weinh). 2025 Jun 5;12(33):e00216. doi: 10.1002/advs.202500216 (PMC12412502; doi:10.1002/advs.202500216)
Supplement: Supplementary file 1 — Supporting Information [file ADVS-12-e00216-s005.docx]

**Figure S1**


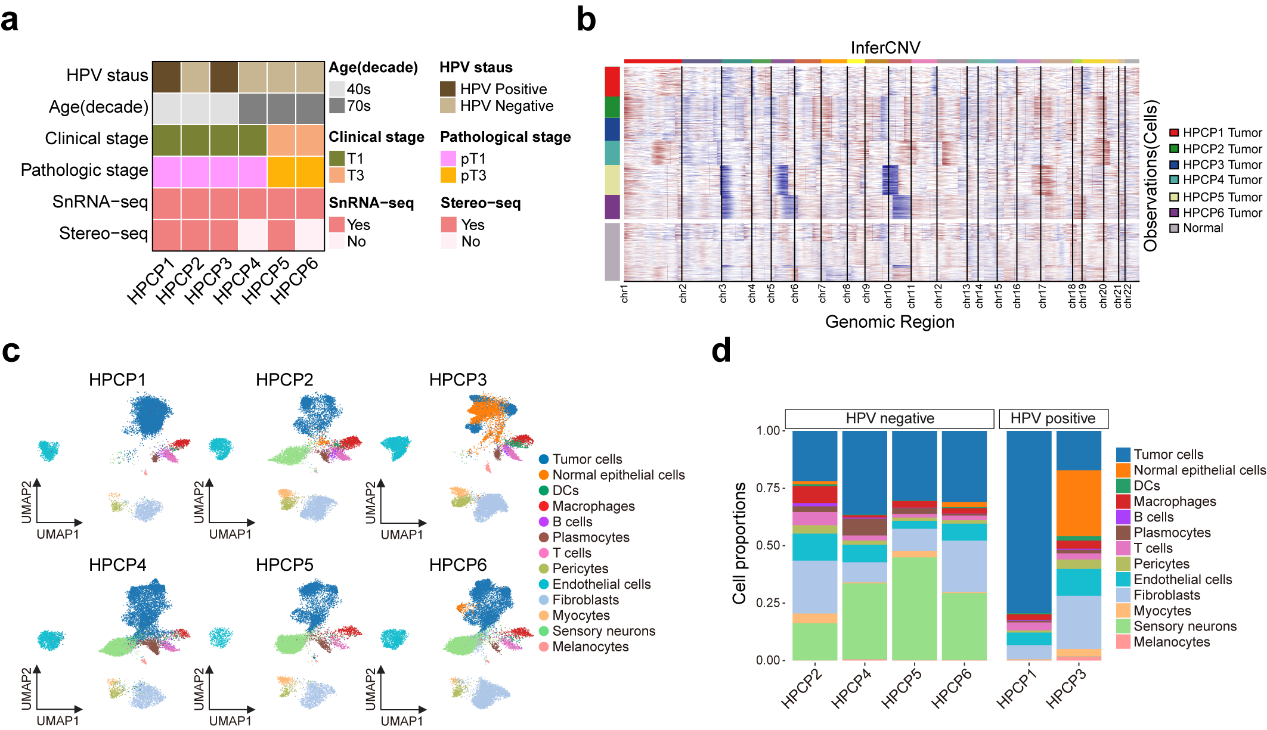


**Figure S1.** Overview of sample characteristics and single-cell transcriptomic analysis. **a,** Heatmap summarizing the sample characteristics, including patient age (grouped by decade), HPV status, clinical stage, pathological stage, and the availability of data types such as snRNA-seq and Stereo-seq. **b,** InferCNV heatmap of epithelial cells derived from snRNA-seq data, highlighting copy number variations (CNVs). The heatmap distinguishes malignant epithelial cells from normal epithelial cells across all patients based on CNV patterns. The upper section displays the CNV profiles of malignant epithelial cells for each patient, highlighting the heterogeneity of malignant epithelium. The lower section shows the CNV stability observed in normal epithelial cells. This analysis reveals genomic alterations that differentiate malignant and normal epithelium. **c,** UMAP plots for each individual patient, with cells color-coded by their respective cell types. **d,** Bar chart illustrating the proportion of each cell type for individual patients, grouped by HPV infection status (HPV-positive and HPV-negative).

**Figure S2**

**
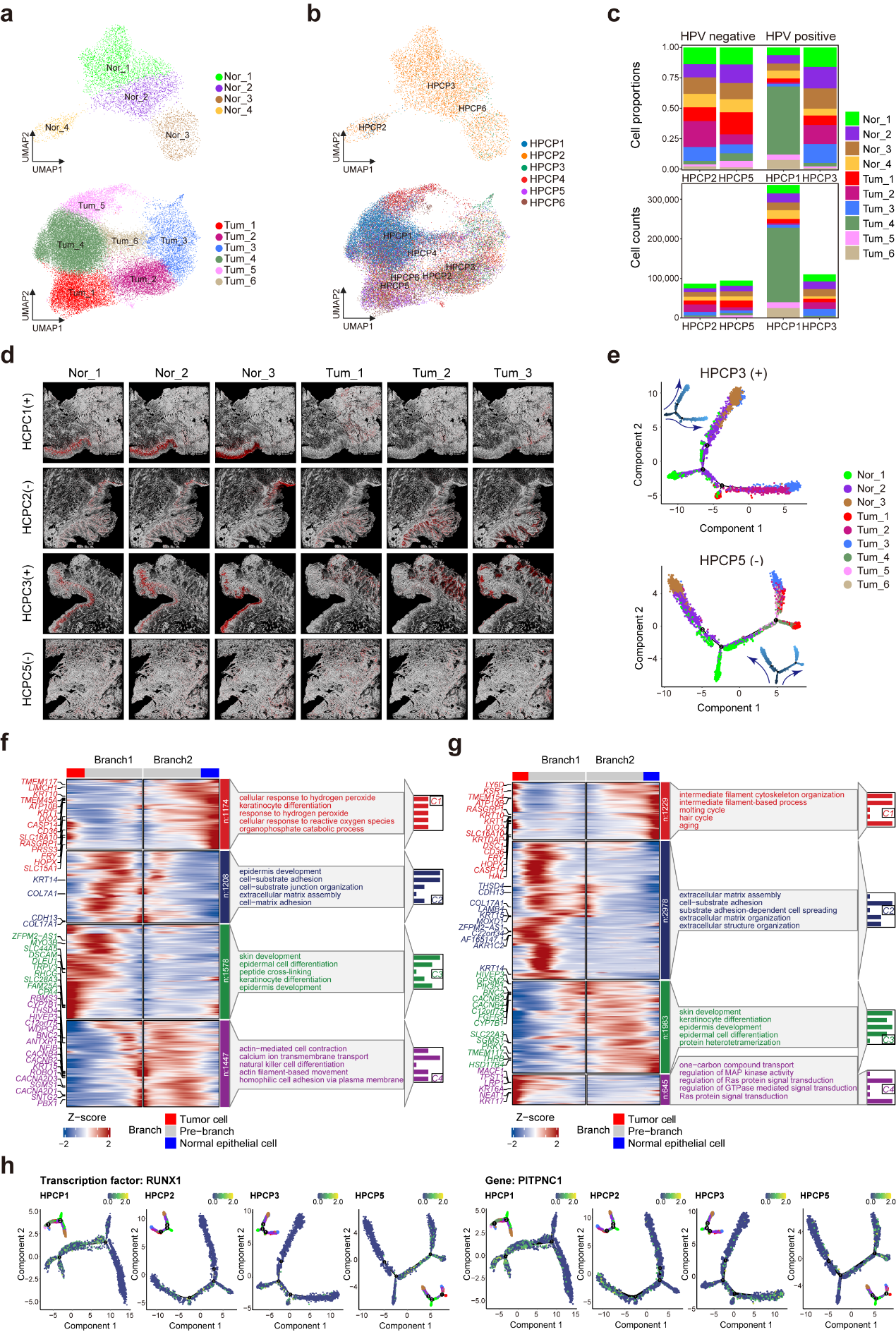
**

**Figure S2**. Evolutionary inference of normal epithelium and tumor cells. **a,** Umap visualizations separately representing normal epithelial cell subtypes and distinct tumor cell subtypes. **b,** Umap visualizations of normal epithelial cell subtypes and tumor cell subtypes, labeled by patient ID. **c,** Stacked bar plot showing the epithelial cell subtype proportion and number in Stereo-seq data, separated by HPV-positive samples and HPV-negative samples. **d,** Spatial distribution of epithelial cell subtypes, mapped onto spatial image individually for all samples. **e,** The pseudo-temporal trajectory among epithelial cell subtypes within the context of HPV infection status is illustrated, distinguishing between HPCP3 (HPV-positive) and HPCP5 (HPV-negative) samples. This visual representation employs a pseudo-temporal path to trace the developmental progression of these cell subtypes. Arrows strategically positioned around pseudo-temporal path serve as guides, delineating the directionality of cellular evolution over time. **f, g,** Pseudo-temporal branch analysis between the two trajectory paths to normal epithelial cells and tumor cells respectively, for HPCP3 sample **(f)** and HPCP5 sample **(g)**. On the left side of the visualization, gene names are annotated to identify the specific markers associated with each branch of the trajectory. The right side is dedicated to annotating enrichment pathways which represent the biological processes along the pseudo-temporal trajectory. **h**, The expression of *RUNX1* and *PITPNC1* along trajectory path for all samples.

**Figure S3**

**
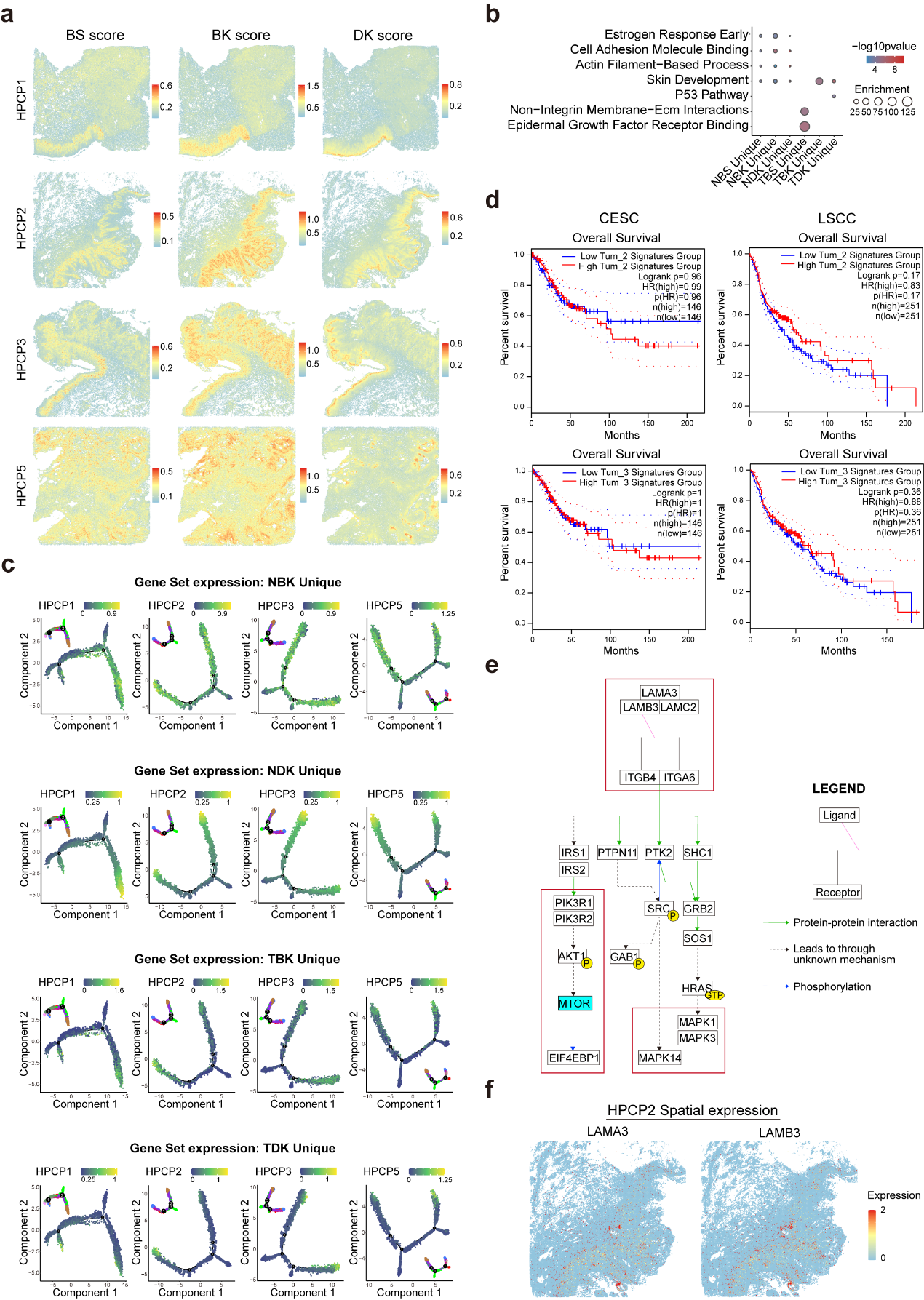
**

**Figure S3**. Features of tumor cell subtypes. **a,** Spatial expression of feature scores (BS, BK, DK) derived from spatial transcriptomics based on marker genes specific to normal epithelium. **b,** Functional enrichment analysis of tissue-specific genes across various stages of tumors and normal epithelium. Color intensity reflects the -log10(p-value) and enrichment scores. **c,** Monocle2 analysis of the evolutionary trajectories of tumor- and normal-epithelium-specific BK and DK gene expression patterns. The expressions of gene sets on the trajectory path of epithelial subtypes are shown for all samples. Zoomed-out trajectory plot is labeled by epithelial cell subtypes. **d,** Survival analysis of high and low expression of Tum_2 and Tum_3 subtype signature genes in CESC and LSSC patients from the TCGA cohort. These survival curves show the prognostic value of Tum_2 and Tum_3 subtype signature genes, statistically tested using the Mantel-Cox log-rank test. **e,** The Laminin-332 signaling pathway involving its binding to ITGA6/ITGB4 heterodimers (refer to <https://www.ndexbio.org>). **f,** Spatial transcriptomic expression of Laminin-332 subunits α3 (LAMA3) and β3 (LAMB3). This panel shows the spatial distribution of these subunits within the tissue, revealing their localized expression patterns in both tumor and normal epithelial regions.


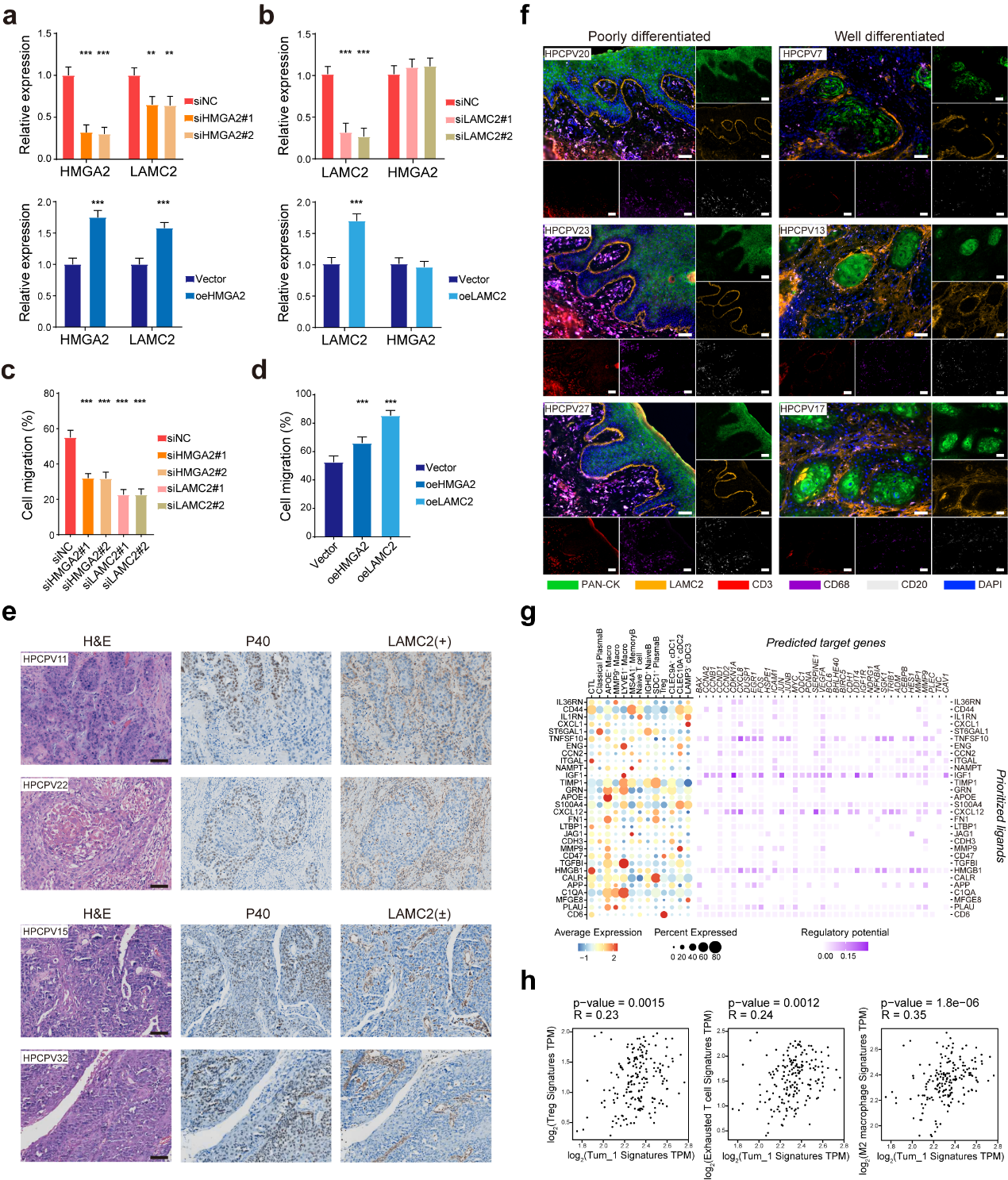
**Figure S4**

**Figure S4**. Tum_1 clinical outcomes and tumor microenvironment characteristics. **a, b,** Quantification of HMGA2 and LAMC2 mRNA levels by quantitative PCR (qPCR) in Penl1 cells after HMGA2 or LAMC2 knockdown (siHMGA2, siLAMC2) or overexpression (oeHMGA2, oeLAMC2) for 72 h, statistically tested using the two-tailed unpaired Student’s t-test.  **c,** **d,** Cell migration assay was performed in Penl1 cells following HMGA2 and LAMC2 knockdown (siHMGA2 and siLAMC2) or overexpression (oeHMGA2 and oeLAMC2) via small interfering RNA and plasmid transfection for 48 hours, statistically tested using the two-tailed unpaired Student’s t-test. **e,** H&E and immunohistochemistry (IHC) staining of penile squamous cell carcinoma. IHC staining images of P40 and LAMC2 respectively standing for PSCC and Tum_1 were presented. LAMC2 shows positive expression in PSCC patients with recurrence, whereas lower-positive expression is observed in patients with relatively favorable outcomes. Scale bar, 100 µm. **f,** Immunofluorescence staining of poorly-differentiated and well-differentiated penile squamous cell carcinoma using antibodies against panCK (green), LAMC2 (yellow), CD3 (red), CD68 (purple), and CD20 (white). Nuclei were counterstained with DAPI (blue). Images were captured using laser confocal microscopy to illustrate the infiltration of immune cells in penile squamous cell carcinoma with varying degrees of differentiation. Scale bars, 50 μm. **g,** Cell-cell communication analysis between immune cells as sender cells and Tumor_1 cells as receiver cells. The left panel shows a dot plot illustrating the expression patterns of ligands in immune cells. The heatmap analyzes the regulatory potential of prioritized ligands from immune cells and predicted target genes in Tumor_1 cells. **h,** Correlation analysis between Tum_1 signature genes and Treg signature genes, between Tum_1 signature genes and T cell signature genes, and between Tum_1 signature genes and M2 macrophage signature genes carried out in ESCC through the TCGA database, statistically tested using the Pearson correlation analysis. Statistical significance is indicated by ***P < 0.001, **P < 0.01.

**Figure S5**

**
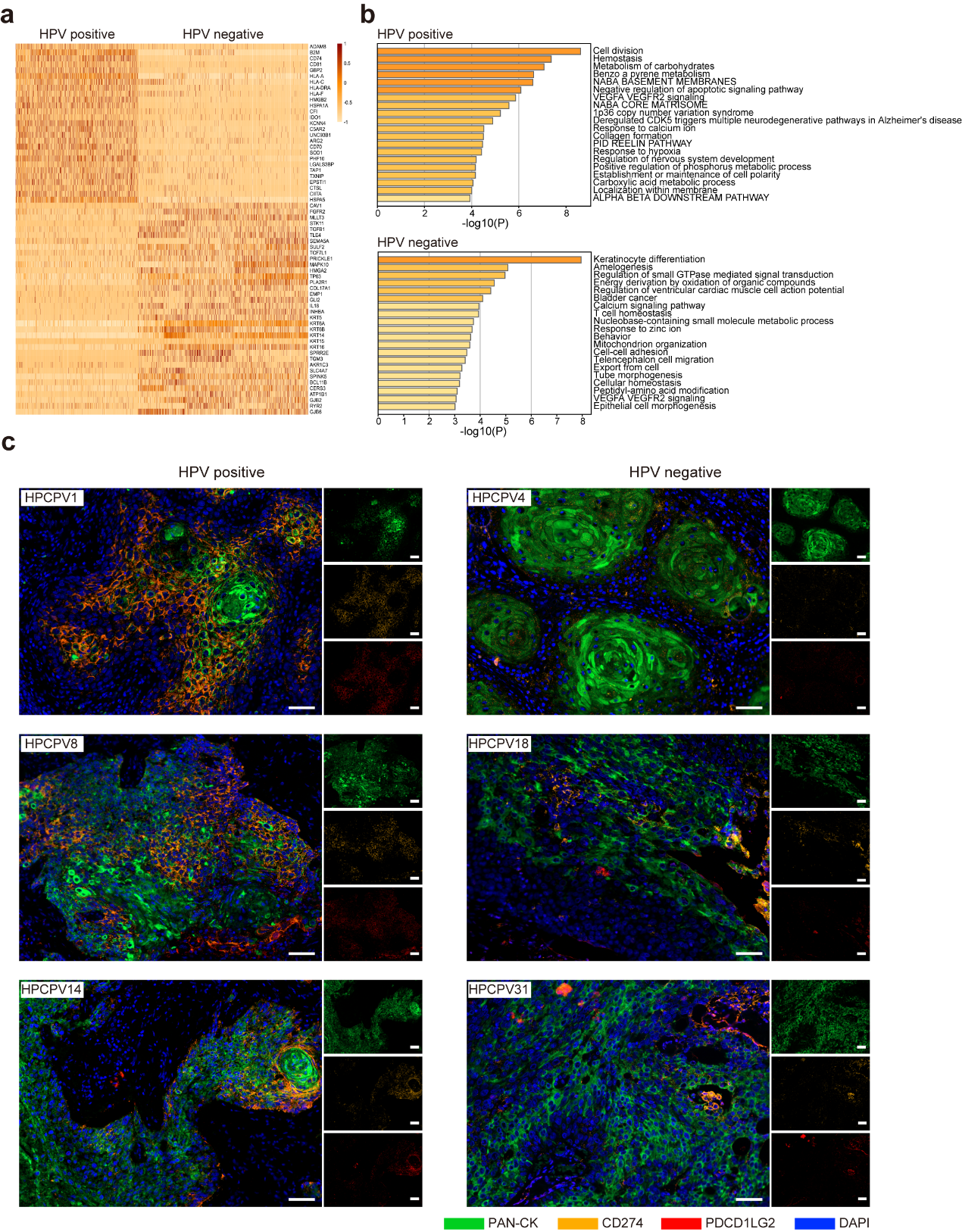
**

**Figure S5**. Genes and pathways related to HPV-positive and HPV-negative tumors. **a,** Differentially expressed genes in HPV-positive and HPV-negative tumors. **b,** Enriched pathways of genes in the trajectory path to HPV-positive tumor and HPV-negative tumor, respectively. **c,** Immunofluorescence staining of penile squamous cell carcinoma using antibodies against panCK (green), CD274 (yellow), PDCD1LG2 (red). Nuclei were counterstained with DAPI (blue). Images were captured using laser confocal microscopy to illustrate the infiltration of immune cells in penile squamous cell carcinoma with varying degrees of differentiation. Scale bars, 50 μm.
