# Supplementary material for: Single‐Cell and Spatial Transcriptomic Profiling of Penile Squamous Cell Carcinoma Reveals Dynamics of Tumor Differentiation and Immune Microenvironment
Source: Adv Sci (Weinh). 2025 Jun 5;12(33):e00216. doi: 10.1002/advs.202500216 (PMC12412502; doi:10.1002/advs.202500216)
Supplement: Supplementary file 2 — Supplementary Table 1 [file ADVS-12-e00216-s001.docx]

| **Patient clinical information** | | | | | | | | **Spatial** | **Single cell** |
| --- | --- | --- | --- | --- | --- | --- | --- | --- | --- |
| **Number** | **SID** | **HPV results** | **Original ID** | **Age** | **Clinical staging** | **Pathologic stage** | **Adjuvant** | **ID2** | **ID3** |
| 1 | HPCP1 | HPV16 | 980755-1 | 46 | T1N0M0 | pT1N0M0 | None | A02776E3 | R231113001,  R231113002 |
| 2 | HPCP2 | / | 980942-5 | 44 | T1N0M0 | pT1N0M0 | None | A02776E4 | R231113003,  R231113004 |
| 3 | HPCP3 | HPV16/HPV51 | 965789-4 | 40 | T1N0M0 | pT1N0M0 | None | A02776E6 | R231113005,  R231113006 |
| 4 | HPCP4 | / | 1007877 | 72 | T1N0M0 | pT1N0M0 | None | / | R231220001,  R231220002 |
| 5 | HPCP5 | / | 1008610-4 | 71 | T3N0M0 | pT3N0M0 | None | A02776F1 | R231220003,  R231220004 |
| 6 | HPCP6 | / | 1008610-6 | 71 | T3N0M0 | pT3N0M0 | None | / | R231220005,  R231220006 |

**Table S1. Integrated spatial omics patient cohort information**

Abbreviation: HPCP, human penile cancer patient; HPV, human papillomavirus
