# Supplementary material for: Single‐Cell and Spatial Transcriptomic Profiling of Penile Squamous Cell Carcinoma Reveals Dynamics of Tumor Differentiation and Immune Microenvironment
Source: Adv Sci (Weinh). 2025 Jun 5;12(33):e00216. doi: 10.1002/advs.202500216 (PMC12412502; doi:10.1002/advs.202500216)
Supplement: Supplementary file 4 — Supplementary Table 3 [file ADVS-12-e00216-s004.docx]

**Table S3. Validation patient cohort information**

| **Number** | **SID** | **Original ID** | **Age** | **Pathologic stage** | **Histology** | **Histology differentiation** | **Adjuvant** | **HPV status** | **Status Last FUP** |
| --- | --- | --- | --- | --- | --- | --- | --- | --- | --- |
| 1 | HPCPV1 | 929226 | 63 | pT1NX | PSCC | Well | None | positive | NED |
| 2 | HPCPV2 | 962497 | 56 | pT2NX | PSCC | Moderately | None | positive | NED |
| 3 | HPCPV3 | 408966 | 74 | pT1NX | PSCC | Moderately | None | negative | NED |
| 4 | HPCPV4 | 1006933 | 57 | pT1NX | PSCC | Well | None | negative | NED |
| 5 | HPCPV5 | 1017393 | 42 | pT2NX | PSCC | Moderately | None | negative | NED |
| 6 | HPCPV6 | 947256 | 60 | pT1N0 | PSCC | Well | None | positive | NED |
| 7 | HPCPV7 | 958575 | 68 | pT1N0 | PSCC | Well | None | negative | NED |
| 8 | HPCPV8 | 951093 | 44 | pT1N0 | PSCC | Moderately | None | positive | NED |
| 9 | HPCPV9 | 945458 | 71 | pT1N0 | PSCC | High-moderately | None | negative | NED |
| 10 | HPCPV10 | 933790 | 70 | pT2NX | PSCC | Moderately | None | negative | NED |
| 11 | HPCPV11 | 932712 | 54 | pT1N0 | PSCC | Well | None | positive | Reccurence |
| 12 | HPCPV12 | 811773 | 63 | pT1N1 | PSCC | Poorly | None | positive | NED |
| 13 | HPCPV13 | 928108 | 59 | pT1N0 | PSCC | Well | None | positive | NED |
| 14 | HPCPV14 | 921998 | 73 | pT3N2 | PSCC | High-moderately | None | positive | NED |
| 15 | HPCPV15 | 909478 | 63 | pT2N0 | PSCC | Moderately | None | negative | NED |
| 16 | HPCPV16 | 889326 | 70 | pT1N2 | PSCC | Well | None | negative | NED |
| 17 | HPCPV17 | 670020 | 65 | pT1N0 | PSCC | Well | None | positive | NED |
| 18 | HPCPV18 | 852607 | 63 | pT1N0 | PSCC | Moderately | None | negative | NED |
| 19 | HPCPV19 | 850848 | 71 | pT2N2 | PSCC | Well | None | negative | NED |
| 20 | HPCPV20 | 807922 | 70 | pT1N0 | PSCC | Poorly | None | negative | NED |
| 21 | HPCPV21 | 792229 | 55 | pT2N1 | PSCC | Well | None | positive | NED |
| 22 | HPCPV22 | 520556 | 69 | pT1N1 | PSCC | Poorly | None | negative | Reccurence |
| 23 | HPCPV23 | 980755 | 46 | pT1N0 | PSCC | Poorly | None | negative | NED |
| 24 | HPCPV24 | 898400 | 57 | pT1NX | PSCC | High-moderately | None | positive | NED |
| 25 | HPCPV25 | 914810 | 59 | pT2N2 | PSCC | Well | None | positive | NED |
| 26 | HPCPV26 | 641633 | 64 | pT1NX | PSCC | Poorly | GP | positive | Reccurence |
| 27 | HPCPV27 | 795273 | 68 | pT1N1 | PSCC | Poorly | None | positive | NED |
| 28 | HPCPV28 | 799226 | 49 | pT1N1 | PSCC | Moderately | TIP | negative | Reccurence |
| 29 | HPCPV29 | 856817 | 64 | pT1N3 | PSCC | High-moderately | PD-1 | positive | Reccurence |
| 30 | HPCPV30 | 856469 | 54 | pT4N1 | PSCC | Well | PF | negative | Reccurence |
| 31 | HPCPV31 | 828547 | 72 | pT1N2 | PSCC | Low-moderately | None | negative | NED |
| 32 | HPCPV32 | 835248 | 44 | pT1NX | PSCC | Low-moderately | None | positive | NED |
| 33 | HPCPV33 | 826913 | 40 | pT1N2 | PSCC | High-moderately | TIP | negative | Reccurence |

Abbreviation: HPCPV, human penile cancer patient for validation; HPV, human papillomavirus; FUP, follow-up; NED, no evidence of disease.
