# Supplementary material for: Single‐Cell and Spatial Transcriptomic Profiling of Penile Squamous Cell Carcinoma Reveals Dynamics of Tumor Differentiation and Immune Microenvironment
Source: Adv Sci (Weinh). 2025 Jun 5;12(33):e00216. doi: 10.1002/advs.202500216 (PMC12412502; doi:10.1002/advs.202500216)
Supplement: Supplementary file 5 — Supplementary Table 4 [file ADVS-12-e00216-s003.docx]

| **Name** | **Catalog** | **Dilution** | **Supplier** |
| --- | --- | --- | --- |
| PAN-CK | ab7753 | 1:200 | Abcam |
| CD4 | ab133616 | 1:200 | Abcam |
| CD8 | sc-1177 | 1:100 | Santa |
| CD68 | ab213363 | 1:200 | Abcam |
| CD20 | ab64088 | 1:200 | Abcam |
| CD274 (PD-L1) | ab282458 | 1:200 | Abcam |
| PDCD1LG2 (PD-L2) | sc-80285 | 1:100 | Santa |
| LAMC2 | ab274376 | 1:200 | Abcam |
| P40 | ab137691 | 1:500 | Abcam |
| LAMC2 | ab210959 | 1:500 | Abcam |

**Table S3. Antibody**
